# Discovery of New Schiff Bases Tethered Pyrazole Moiety: Design, Synthesis, Biological Evaluation, and Molecular Docking Study as Dual Targeting DHFR/DNA Gyrase Inhibitors with Immunomodulatory Activity

**DOI:** 10.3390/molecules25112593

**Published:** 2020-06-02

**Authors:** Ashraf S. Hassan, Ahmed A. Askar, Ahmed M. Naglah, Abdulrahman A. Almehizia, Ahmed Ragab

**Affiliations:** 1 Organometallic and Organometalloid Chemistry Department, National Research Centre, Dokki 12622, Cairo, Egypt; 2 Botany and Microbiology Department, Faculty of Science (Boys), Al-Azhar University, Nasr City, Cairo, Egypt; 3 Department of Pharmaceutical Chemistry, Drug Exploration and Development Chair (DEDC), College of Pharmacy, King Saud University, Riyadh 11451, Saudi Arabia; anaglah@ksu.edu.sa (A.M.N.); mehizia@ksu.edu.sa (A.A.A.); 4 Peptide Chemistry Department, National Research Centre, Dokki 12622, Cairo, Egypt; 5 Department of Pharmaceutical Chemistry, College of Pharmacy, King Saud University, Riyadh 11451, Saudi Arabia; 6 Chemistry Department, Faculty of Science (Boys), Al-Azhar University, Nasr City, Cairo, Egypt

**Keywords:** Schiff bases, pyrazole moiety, antimicrobial, antiproliferative, enzyme inhibitor, DHFR, DNA gyrase, molecular docking

## Abstract

A series of *Bis*-pyrazole Schiff bases (**6a**–**d** and **7a**–**d**) and mono-pyrazole Schiff bases (**8a**–**d** and **9a**–**d**) were designed and synthesized through the reaction of 5-aminopyrazoles **1a**–**d** with aldehydes **2**–**5** using mild reaction condition with a good yield percentage. The chemical structure of newly formed Schiff bases tethered pyrazole core was confirmed based on spectral and experimental data. All the newly formed pyrazole Schiff bases were evaluated against eight pathogens (Gram-positive, Gram-negative, and fungi). The result exhibited that, most of them have good and broad activities. Among those, only six Schiff bases (**6b**, **7b**, **7c**, **8a**, **8d**, and **9b**) displayed MIC values (0.97–62.5 µg/mL) compared to Tetracycline (15.62–62.5 µg/mL) and Amphotericin B (15.62–31.25 µg/mL), MBC values (1.94–87.5 µg/mL) and selectivity to tumor cell than normal cells. Immunomodulatory activities showed that the promising Schiff bases increase the immunomodulator effect of defense cell and the Schiff base **8a** is the highest one by (Intra. killing activity = 136.5 ± 0.3%) having a pyrazole moiety as well as amide function (O=C-NH_2_) and piperidinyl core. Furthermore, the most potent one exhibited broad activity depending on both MIC and MBC values. Moreover, to study the mechanism of these pyrazole Schiff bases, two active Schiff bases **8a** and **9b** from six derivatives were introduced to study the enzyme assay as dihydrofolate reductase (DHFR) on *E. coli* organism and DNA gyrase with two different organisms, *S. aureus* and *B. subtilis*, to determine the inhibitory activities with lower values in the case of DNA gyrase (**8a** and **9b**) or nearly as DHFR compound **9b**, while pyrazole **8a** showed excellent inhibitory against all enzyme assay. The molecular docking study against dihydrofolate reductase and DNA gyrase were performed to study the binding between active site in the pocket with the two Schiff bases (**8a** and **9b**) that exhibited good binding affinity with different bond types as H-bonding, aren-aren, and arene-cation interaction as well as study the physicochemical and pharmacokinetic properties of the two active Schiff bases **8a** and **9b**.

## 1. Introduction

One of the most common causes of death all over the world is the microbial infections and multidrug-resistant bacteria. According to the World Health Organization (WHO), the resistance of microbes to antibiotics drugs is one of the dangerous health problems that threaten humans. Therefore, the production of novel bioactive compounds acting as antimicrobial agents for overcoming the resistance problem is an urgent topic that configures interest between the organic and medicinal chemistry researchers and is considered one of the greatest achievements over time [1,2,3]. Antibiotic-resistant infection problem arises from the broad use and abuse of conventional antibiotics, already, every year an estimated 700,000 people around the world die of drug-resistant diseases, 10 million people could die each year from diseases that have grown resistant [4,5,6].

Schiff bases (–CH=N– function) have the biological and pharmacological applications [7,8,9]. Schiff base (**I**), 4-chloro-2-((4-fluorobenzyl imino)methyl)phenol, showed antibacterial activity against *E. coli* and *P. fluorescence* [10], as well as Schiff base (**II**), bearing 2-(piperazin-1-yl)ethan-amine derivative, exhibited good anticancer activity against lung (NCI H-522) cells [11]. Moreover, Schiff base (**III**) with quinoline derivative as an active core demonstrated in vitro anti-inflammatory activity [12]. One of the most wide pharmacophore cores is pyrazole derivatives, and our study involves the design and synthesis of new pyrazole derivatives. 

**Figure 1:**
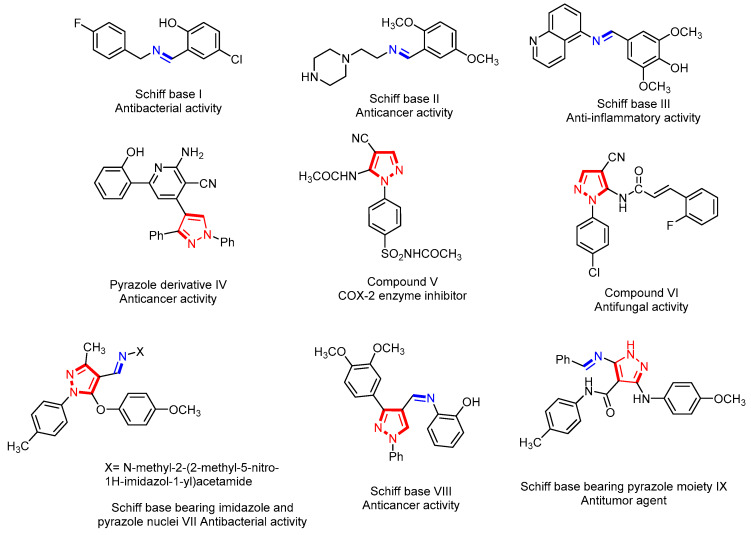
Structures of bioactive Schiff bases and pyrazole compounds **I**–**IX**.

Besides, literature displayed many pyrazoles that have a wide array of biological activities such as neuraminidase inhibitors against influenza H1N1 virus [13], apoptotic inducers [14], antimicrobial, cytotoxic [15], and antimalarial [16] activities. Pyrazole moiety attached to enaminonitrile pyridine derivative (**IV**) showed anticancer activity against both HePG-2 and MCF-7 lines [17]. Also, pyrazole with *N*-(4-phenyl)sulfonylacetamide derivative (**V**) exhibited good activity and selectivity toward the COX-2 enzyme [18]. On the other hand, pyrazole moiety containing both *N*-(4-chlorophenyl) and (2-fluorophenyl)acrylamide derivative (**VI**) produced potent antifungal activity [19]. Recently, there is a growing interest in the synthesis of Schiff bases tethered pyrazole ring because of their importance in biological activities [20,21,22]. For example, Schiff base (**VII**), bearing imidazole and pyrazole nucleus, showed potency against *E. coli* [23]. While, Schiff base (**VIII**), derived from *N*-phenyl-pyrazole derivative with 2-aminophenol, reveled promising anticancer activity close to doxorubicin [24]. Furthermore, we have reported the synthesis of Schiff base bearing pyrazole moiety (**IX**), 5-(benzylideneamino)-3-(4-methoxyphenylamino)-*N*-(4-methylphenyl)-1*H*-pyrazole-4-carboxamide, displayed a potent antitumor agent against MCF-7 lines [25] (Figure 1). 

Dihydrofolate reductase (DHFR) enzyme is a target for several anticancer and antibacterial drugs and of high importance in medicinal chemistry [26,27] because of its function as a cofactor in the biosynthesis of nucleic acids and amino acids. Mechanism of action for DHFR is blocking the synthesis of DNA, RNA, and proteins, causing cell growth arrest [28,29]. Also, DNA gyrase is an enzyme that belongs to type II topoisomerases and catalyzes changes in DNA topology [30]. In addition, it is composed of two chains GyrA and GyrB subunits that are responsible for the transient break of two strands of DNA, as well as introducing negative supercoils in DNA during replication. Drugs that target DNA gyrase exhibited their antibacterial activity by two mechanisms as gyrase poisoning as in Ciprofloxacin or by blocking the ATP binding site as in Novobiocin [31] and depending on the previous important function, it has become an attractive target for the development of antibacterial drug [32]. Therefore, DHFR and DNA topoisomerases have a proven track record in supporting their role in microbial diseases and cancer chemotherapy [33,34]. The immune system is a remarkably advanced vertebrate defense system and its main function is defense against invaders by producing cell and molecular varieties that can recognize and thus eliminate unlimited varieties of foreign and harmful agents [35,36]. In general, immunomodulators can be classified as two different types depending on their effects: immunosuppressants and Immunostimulants, and both could mount an immune response or defend against pathogens or tumors [37]. Immunomodulation is the process produced by the administration of a drug or compound to alter an immune response positive or negative. A number of proteins, amino acids, and natural compounds showed an important ability to regulate immune responses, including interferon-γ (IFN–γ) steroids [38].

For the aforementioned reasons biological activities of Schiff bases, pyrazole moiety and Schiff bases tethered pyrazole ring and in continuation of our research program [39,40,41,42,43,44,45,46,47,48,49,50], in this work, we have synthesized a new series of *bis*-pyrazole Schiff bases (**6a**–**d** and **7a**–**d**) and mono-Schiff bases tethered pyrazole moiety (**8a**–**d** and **9a**–**d**) for the examination of their in vitro antimicrobial, antiproliferative, immunomodulatory activities and extend our work to drug resistance activity (MDRB), enzymes assessment (dihydrofolate reductase and DNA gyrase), as well as some of in silico studies as physicochemical properties, structure-activity relationship, and the molecular docking hoping to find new pyrazole derivatives as dual-targeting DHFR and DNA gyrase inhibitors. 

## 2. Results and Discussion

### 2.1. Chemistry

**Scheme 1:**
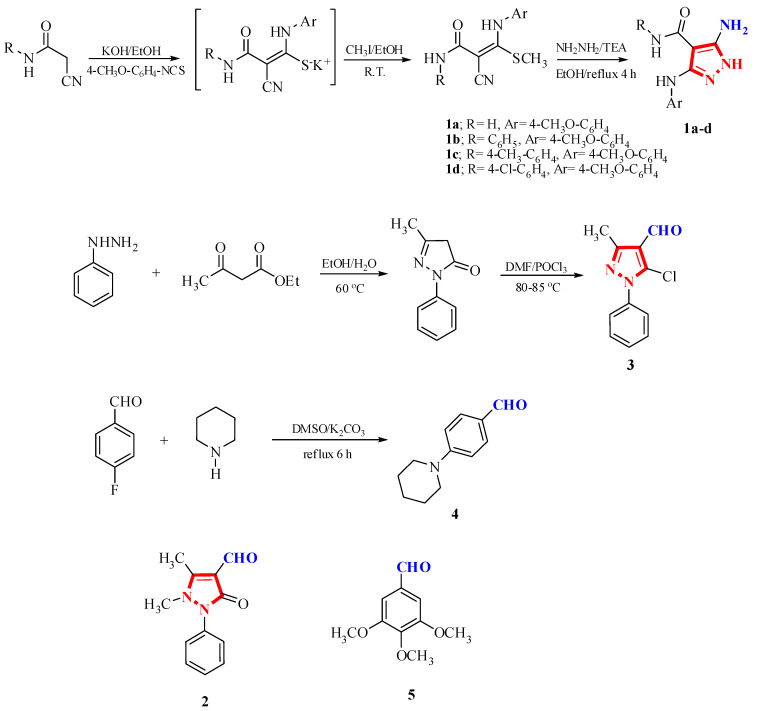
The synthesis of starting materials (**1a**–**d**, **3** and **4**) and the structure of (**2** and **5**).

The starting material, 5-amino-*N*-aryl-3-(4-methoxyphenylamino)-1*H*-pyrazole-4-carboxamide **1a**–**d,** was prepared according to the reported method [51,52,53]. New Schiff bases with hybrid active core pyrazole derivatives were synthetized by the reaction of pyrazole derivatives **1a**–**d** with different aldehyde containing different pharmacophore atoms or groups that are represented in Scheme 1 namely 1,5-dimethyl-3-oxo-2-phenyl-2,3-dihydro-1*H*-pyrazole-4-carbaldehyde (**2**), 5-chloro-3-methyl-1-phenyl-1*H*-pyrazole-4-carbaldehyde (**3**), 4-(piperidin-1-yl)benzaldehyde (**4**), and 3,4,5-trimethoxybenzaldehyde (**5**) hoping to be effective as antimicrobial and anticancer activities.

**Scheme 2:**
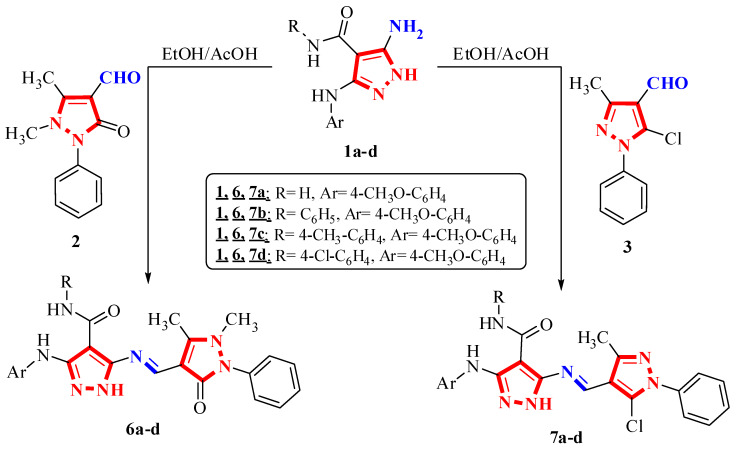
The synthesis of *Bis*-pyrazole Schiff bases (**6a**–**d** and **7a**–**d)**.

The newly designed *bis*-pyrazole Schiff bases **6a**–**d** or **7a-d** were prepared via the condensation of 5-aminopyrazole derivatives **1a**–**d** with 1,5-dimethyl-3-oxo-2-phenyl-2, 3-dihydro-1*H*-pyrazole-4-carbaldehyde **(2)** or 5-chloro-3-methyl-1-phenyl-1*H*-pyrazole-4-carbaldehyde (**3**), respectively (Scheme 2). The structures of *bis*-pyrazole Schiff bases **6** and **7** were proved on the basis of analytical and spectral data. IR spectrum of 5-((1,5-dimethyl-3-oxo-2-phenyl-2,3-dihydro-1*H*-pyrazol-4-yl)methylene-amino)-3-(4-methoxyphenylamino)-1*H*-pyrazole-4-carboxamide (**6a**) confirmed the presence of absorption bands at 3359, 3165, 1675, and 1654 cm^−1^ for NH, NH_2,_ C=O (antipyrine) and C=O (amide) groups, respectively. Its ^1^H NMR (400 MHz) spectrum showed singlet signals attributable to the protons of -CH=N- and 2NH at *δ* 8.68, 8.85, and 12.37 ppm, respectively. In addition to three singlet signal related to -CH_3_, -N-CH_3_, and -OCH_3_ protons appeared at *δ* 2.61, 3.37, and 3.70 ppm, respectively. The protons of aromatic rings appear as two doublet at *δ* 6.85 and 7.37 ppm for four aromatic protons with coupling constant (*J*) 8.2 and 7.4 Hz, as well as, the five protons that exist as multiplet in region *δ* 7.45–7.48 ppm for three protons and triplet signal at 7.56 ppm for the last two protons. Furthermore, two singlet singlets at *δ* 7.19 and 7.80 ppm for NH_2_-amide function. 

Also, its ^13^C NMR (101 MHz) spectrum showed the characteristic signals for CH_3_, -N-CH_3_ and OCH_3_ at *δ* 11.50, 33.93, and 55.21 ppm, respectively. The carbons for the carbonyl groups of amide function and antipyrine moiety appeared at *δ* 162.54 and 166.76 ppm, respectively. The mass spectrum showed its [M^+^] peak (*m*/*z* 445) corresponding to C_23_H_23_N_7_O_3_ (molecular formula) with the relative intensity = 13.44%. 

**Scheme 3:**
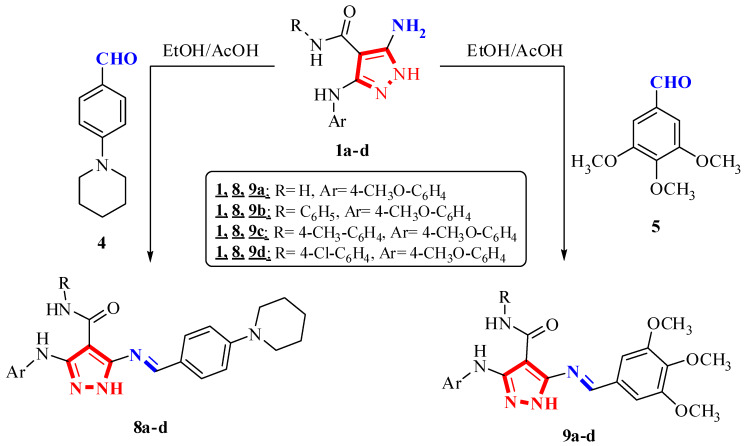
The synthesis of Schiff bases tethered mono-pyrazole moiety (**8a**–**d** and **9a**–**d**).

Also, the synthesis of Schiff bases tethered pyrazole moiety **8a**–**d** or **9a**–**d** were performed by the condensation of 5-aminopyrazole derivatives **1a**–**d** with 4-(piperidin-1-yl) benzaldehyde (**4**) or 3, 4, 5-trimethoxybenzaldehyde (**5**), respectively, in good yield (Scheme 3). The ^1^H NMR spectrum of compound **8b** revealed four singlet signals at *δ* 8.71, 8.77, 10.14, and 12.56 ppm for the protons of -CH=N- and 3NH groups, respectively. The protons of the methoxy group appeared at *δ* 3.72 ppm as a singlet signal. Moreover, the piperidine moiety protons appeared at *δ* 1.60 ppm for six protons as a broad signal and 3.43 ppm for four protons. The aromatic protons appear in region *δ* 6.89–7.85 ppm for thirteen protons and represented as three doublet, one triplet, and one multiplet signals at *δ* 6.89(d), 7.68(d), 7.85(d), 7.08 (t), and 7.35–7.48 (m) ppm respectively, with coupling constant (*J*) ranging between 8.1–8.3 Hz. Its ^13^C NMR spectrum showed three characteristic signals at *δ* 23.95, 24.93, and 47.65 ppm referred to as the carbons of piperidinyl moiety. In addition, two signals at *δ* 55.20 and 163.06 ppm for the carbons of -OCH_3_ and C=O, respectively, as well as aromatic carbons and C=N appeared from *δ* 92.57 to 160.09 ppm. The mass spectrum exhibited the molecular ion peak at *m*/*z* = 494 [M^+^, 32.82%] corresponding to C_29_H_30_N_6_O_2_ (molecular formula).

In the same way, treatment of 5-aminopyrazole derivative **1c** with 3,4,5-trimethoxybenzaldehyde (**5**) afforded 3-(*p*-methoxyphenylamino)-*N*-*p*-tolyl-5-(3,4,5-trimethoxybenzylideneamino)-1*H*-pyrazole-4-carboxamide (**9c**) having four methoxy groups, as well as *N*-*p*-tolyl 4-carboxamide based on elemental analysis and spectral data. IR spectrum of Schiff base **9c** showed characteristic bands related to NH and carbonyl groups at 3437, 3285, and 1655 cm^−1^, respectively. The ^1^H NMR spectrum of Schiff base **9c** exhibited four singlet signals at *δ* 2.27, 3.73, 3.79, and 3.92 ppm for one methyl and four methoxy groups. Furthermore, four singlet signals at upfield can be classified as the signal at *δ* 8.70 ppm related to -CH=N and three singlet signals at *δ* 8.96, 9.95, and 12.64 ppm for three NH protons as well as ten aromatic protons between *δ* 6.91 to 7.59 ppm. The ^13^C NMR spectrum of **9c** displayed a singlet signal at 21.31 ppm for methyl group and three singlet signals at 55.04, 56.79, and 60.65 related to four methoxy groups as well as one signal at 162.88 for carbonyl group beside aromatic carbons that appeared in region 91.93–161.74 ppm.

### 2.2. Biological Evaluation

#### 2.2.1. In Vitro Antimicrobial Activities

Antimicrobial activities of the newly formed Schiff bases (**6a**–**d, 7a**–**d, 8a**–**d,** and **9a**–**d**) and reference drugs were evaluated against three Gram-positive, three Gram-negative, and two fungi strain at Al-Azhar University, Cairo, Egypt. The inhibition zones (IZ, in mm ± standard deviation) and the minimal inhibitory concentration (MIC) (μg/mL) were determined by the conventional paper disk diffusion method [54,55]. The broad-spectrum antibiotics, Tetracycline and Amphotericin B were used as positive controls. The results are presented in Table 1, Table 2 and Table 3.

From the inhibition zone measurements (IZ, Table 1), we can conclude that there are six Schiff bases (**6b**, **7b**, **7c**, **8a**, **8d**, and **9b**) that displayed inhibition zone more than or near to the reference drugs (Tetracycline and Amphotericin B) against pathogenic microbes. For Gram positive and Gram negative, it was found that most of the Schiff bases exhibited moderate to potent anti-bacterial activities with inhibition zone (IZ) ranging between 12 and 33 mm for all pathogen tested in this study compared to Tetracycline that have IZ ranging between 20 and 25 mm. Moreover, most of the newly synthesized Schiff bases showed IZ between 9 and 27 mm, in comparison to Amphotericin B as a therapeutic abroad anti-fungal agent (18–22 mm). This result encouraged us to complete the study and measure the minimal inhibitory concentrations (MIC, µg/mL) of the potent Schiff bases (**6b**, **7b**, **7c**, **8a**, **8d**, and **9b**). The results are represented in Table 2 and Table 3.

From Table 2 and Table 3**,** the six most potent Schiff bases demonstrated broad, potent, and excellent activities against *B. subtilis* (Bs), where **6b** (7.81 µg/mL, 4-fold), **7b** (5.57 µg/mL, 5-folds), **7c** (9.25 µg/mL, 3-folds) **8a** (1.92 µg/mL, 16-folds), **8d** (3.9 µg/mL, 8-folds), and **9b** (4.5 µg/mL, 6-folds) were more potent compared to Tetracycline (31.25 µg/mL, standard antibacterial drug). The two *Bis*-pyrazole Schiff bases (**7b** and **7c**) have only one difference in their structure by replacing phenyl (C_6_H_5_-) with 4-methylphenyl (4-CH_3_-C_6_H_4_-). The result showed good activities in comparison to standard drugs against both Gram positive and negative strains. The presence of phenyl ring in **7b** makes it more effective than **7c** (4-methyl phenyl ring), where a Schiff base **7b** exhibited 32-folds (1.95 µg/mL), 16-fold (3.9 µg/mL), and 8-folds (3.9 µg/mL) more potent activity than that of Tetracycline (62.5, 62.5, and 31.25 µg/mL) against *S. aureus* (Sa), *E. faecalis* (Ef), and *S. typhi* (St), respectively. Furthermore, Schiff base **7b** showed comparable activities toward both fungal strains used in this study with MIC values (15.62, 31.25 µg/mL) against *C. albicans* (Ca) and *F. oxysporum* (Fo), respectively, with two-folds higher than Schiff base **7c**. 

Schiff base **8a** bearing amide group in position four of pyrazole moiety exhibited minimal inhibitory concentrations (0.97 and 5.57 µg/mL) with 16 and 11.2-folds more than Tetracycline (15.62 and 62.5 µg/mL) against *E. coli* (Ec) and *P. aeruginosa* (Pa), respectively. From Table 3, among the tested pyrazole Schiff bases that exhibited anti-fungal activities, the two Schiff bases **8a** and **9b** revealed the best antifungal results with MIC values (7.81 and 15.62 µg/mL) against *C. albicans* (Ca) and *F. oxysporum* (Fo), respectively, that reveled 2-fold activity in comparison with Amphotericin B (15.62, 31.25 µg/mL).

#### 2.2.2. Minimal Bactericidal Concentration (MBC) and Minimum Fungicidal Concentration (MFC)

The MBC is complementary to the MIC; whereas the MIC test demonstrates the lowest level of antimicrobial agent that greatly inhibits growth, but the MBC determines the lowest level of antimicrobial agent that leads to the death of microbial organisms. In other words, if a MIC shows inhibition only, i.e., the antimicrobial activity does not cause death, in contrast to MBC that causes death [56]. MBC is usually presented as MBC_50_, which means the drug concentration kills 50% of the initial bacterial population [57]. The promising pyrazole Schiff bases **6b**, **7b**, **7c**, **8a**, **8d**, and **9b** showed bactericidal activities in general. For Gram positive bacteria, MBC values ranged between (3.7 and 53.12 µg/mL) compared to Tetracycline (40.62 and 93.75 µg/mL), and the most active Schiff base **8a** exhibited bactericidal activity (3.9, 5.57, 6.63 µg/mL) for *B. subtilis* (Bs), *S. aureus* (Sa), *E. faecalis* (Ef) compared to Tetracycline (40.62, 87.5, 93.75 µg/mL) respectively, followed by **9b** and **7c** that showed MBC values (9.2, 15.62, 7.41 µg/mL) and (10.58, 3.7, 6.63 µg/mL). Also, three Schiff bases **8a**, **9b**, and **7c** revealed MBC values (1.94, 14.05, 12.49 µg/mL) and (10.58, 31.25, 59.37 µg/mL) for both *E. coli* (Ec) and *P. aeruginosa* (Pa), respectively. 

On the other hand, for Gram negative bacteria, Schiff base **7b** showed the best MBC value among the six Schiff bases (6.63 µg/mL with nearly 6.6 folds potent than the standard drug) against *S. typhi* (St). But, Schiff base **7c** exhibited the worst results to Gram negative bacterial strains. In the same way, four from six of the promising Schiff bases known as **7b**, **8a**, **8d**, and **9b** showed a good fungicidal activity with MFC values ranging from (12.94–56.25 µg/mL) in comparison with Amphotericin B (34.62–65.62 µg/mL). However, both **6b** and **7c** showed slightly increased activity than reference drug.

Previous studies reported that there is a relation between the MIC and MBC to decide whether the newly designed compounds have cidal or statical activities for bacteria and fungi [58]; antibacterial agent that has MBC/MIC ratios higher than or equal to 8 is considered as a bacteriostatic agent, and by contrast, the MBC/MIC ratio between 1 and 2 is regarded as bactericidal agent according to Clinical and Laboratory Standards Institute (CLSI) standards [59,60]. As shown in Table 2 and Table 3 and by applying MBC/MIC and MFC/MIC ratios, the values obtained ranged between one and nearly two and that expressed all the promising compounds show bactericidal and fungicidal activities with lower concentration depending on MBC, MFC, and MIC values.

#### 2.2.3. In Vitro Antiproliferative Activities

**Figure 2:**
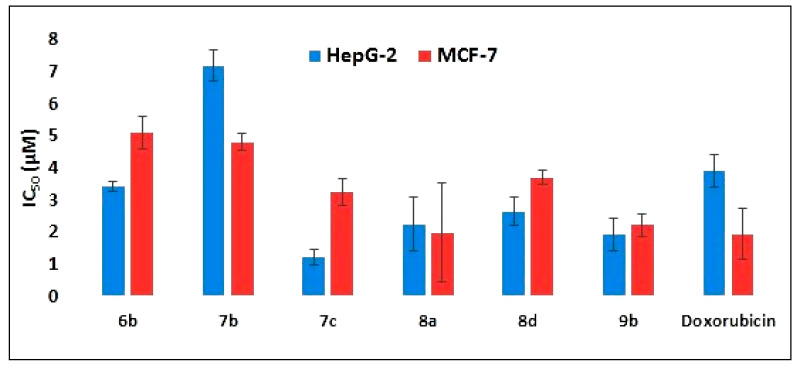
The antiproliferative activities (IC_50_, µM) of the Schiff bases and doxorubicin against HepG-2 and MCF-7 cell lines.

Our work is extended to study *in vitro* antiproliferative activities (IC_50_, µM) of the most potent Schiff bases **6b**, **7b**, **7c**, **8a**, **8d**, and **9b** that are tested against two human cancer cell lines namely hepatocellular carcinoma cell line (HepG2) and mammary gland breast cancer cell line (MCF-7) as well as normal, non-cancer cells (Vero cells, ATCC CCL-81) according to the 3-[4,5-dimethyl-2-thiazolyl)-2,5-diphenyl-2*H*-tetrazolium bromide (MTT) protocol [61,62,63], in comparison with that of doxorubicin as a well-known chemotherapeutic agent (positive control) and the results are shown in Table 4 and Figure 2. 

From Table 4 and Figure 2 results, it was observed that most of the Schiff bases displayed potent cytotoxicity with low micromolar (µM) concentration. For HepG-2 lines, five Schiff bases **6b**, **7c**, **8a**, **8d**, and **9b** expressed IC_50_ lower than doxorubicin and their cytotoxicity ranged between (1.22 ± 0.23 and 3.42 ± 0.17 µM) in comparison to reference drug (3.92 ± 0.50 µM), only Schiff base **7b** showed lower toxicity with (0.54-fold) against doxorubicin. Notably, three Schiff bases **7c**, **8a,** and **9b** exhibited remarkable antitumor activities with IC_50_ values 1.22 ± 0.23, 2.25 ± 0.85, and 1.92 ± 0.49, respectively.

On the other hand, the antiproliferative activities of the Schiff bases against MCF-7 lines showed moderate activities except for the two Schiff bases **8a** (1.98 ± 1.55 µM, 0.979 fold) and **9b** (2.21 ± 0.36 µM, 0.877 fold) which showed values near to doxorubicin (1.94 ± 0.80 µM). Nevertheless, other derivatives exhibited only moderate activities. 

To determine the safety and selectivity of the most potent Schiff bases **6b**, **7b**, **7c**, **8a**, **8d**, and **9b**, the cytotoxicity of promising compounds was determined on healthy non-cancer cells (Vero cells, ATCC CCL-81) from an African green monkey kidney continuous cell by colorimetric MTT assay. The new Schiff bases showed very lower toxicity on healthy non-cancer cells Vero cells with (IC_50_ > 120), and that proved that these new Schiff bases **6b**, **7b**, **7c**, **8a**, **8d**, and **9b** displayed safety and revealed selectivity toward cancerous cells. 

Finally, from all the previously studied and because of the efficiency of the promising six Schiff bases as antimicrobial and antiproliferative potential agents, these derivatives were chosen for further investigation.

#### 2.2.4. Immunomodulatory Activity

In this section, we gain insight into the *in vitro* immunomodulatory potential of the active compounds. Innate immunity is the first line of defense against pathogens present in our environment such as fungi, bacteria, and viruses and eliminates damaged cells and tumor cells characterized by rapid response, infection halting. The innate immunity system may include macrophage, mast cells, dendritic cells (DCs), eosinophils, basophils, neutrophils as well as invariant natural killer cells (NK cells) [64]. The neutrophil can cause microorganisms intracellular killing [65]. The immunomodulatory activity expressed by percentage (%) values for the intracellular killing was performed using nitro blue tetrazolium (NBT) reductase assay, where the increasing percentage is related to improvement in the killing ability of the neutrophils, and was performed according to the reported method [66]. The results are represented in Table 5. 

The potent Schiff bases **6b**, **7b**, **7c**, **8a**, **8d,** and **9b** were evaluated as immunomodulatory agents by percentage values that ranged between (49.6 ± 0.14%) and (136.5 ± 0.3%) and depending on the obtained results the pyrazole derivatives generally induced the immune system to defend against pathogens or tumors by variable percentage. The highest immunostimulatory action was shown by compound **8a** (136.5 ± 0.3%) that has a pyrazole moiety as well as amide function (O=C-NH_2_) and piperidinyl core followed by compound **9b** with a ratio (115.2 ± 0.5%) having pyrazole and benzamide (Ph-NH-C=O) in position four in addition to three methoxy groups.

#### 2.2.5. Drug Resistance

The synthesized pyrazole Schiff bases **6b**, **7b**, **7c**, **8a**, **8d**, and **9b** were tested for their in vitro antibacterial activity against a panel of multidrug-resistant bacteria (MDRB) classified as one clinical strain *S. aureus* (ATCC 43300) (MRSA) with three standard strain *S. aureus* (ATCC 33591), *E. coli* (ATCC BAA-196), and *P. aeruginosa* (ATCC BAA-2111). The broad-spectrum antibiotic Norfloxacin was used as a positive control. The obtained results from Table 6 revealed that the synthesized pyrazole Schiff bases showed varying degrees of activities with strong activities (inhibition zone higher than 15 mm) against the tested microorganisms and therefore, MIC and MBC were performed and are shown in Table 7.

From Table 7**,** it is observed that the MIC values range, for the most promising six Schiff bases **6b**, **7b**, **7c**, **8a**, **8d**, and **9b**, between 1.95 and 15.62 µg/mL as well as MBC values between 3.31 and 31.25 µg/mL against MDRB strains compared with Norfloxacin MIC (0.78–3.13 µg/mL) and MBC (1.56–4.69 µg/mL). Most of the tested pyrazole Schiff bases **6b**, **7b**, **7c**, **8a**, **8d**, and **9b** showed remarkable broad-spectrum activities against both Gram-positive and Gram-negative bacteria for inhibitory or bactericidal activity and among them Schiff bases **8a** and **9b** showed the highest activities in comparison with other derivatives; compound **8a** with mono-pyrazole, piperidinyl, and *p*-tolyl-amide derivative demonstrated MIC values ranging between (1.95 and 7.8 µg/mL). Besides, Schiff base **9b** having a mono pyrazole nucleus and benzamide in position four also showed MIC values (1.95–6.25 µg/mL) and MBC values (3.31–11.87 µg/mL). According to the Clinical and Laboratory Standards Institute (CLSI) standards and by applying the MBC/MIC ratio, we found that all the tested pyrazole Schiff bases exhibited values less than two, meaning bactericidal property.

#### 2.2.6. Enzyme Assessment of Dihydrofolate Reductase (DHFR) and DNA Gyrase 

**Figure 3:**
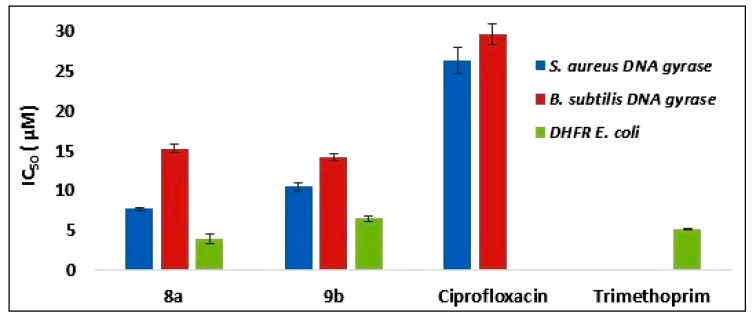
The inhibitory activities (IC_50_, µM) of the two Schiff bases (**8a** and **9b**) and reference drugs against DNA gyrase and dihydrofolate reductase (DHFR).

In continuation of our previous efforts, we targeted the most two active Schiff bases (**8a** and **9b**) depending on previous studies as antimicrobial and anticancer agents from the previous results to evaluate their activities and mechanism against DHFR and DNA gyrase enzymes. The two active Schiff bases (**8a** and **9b**) were introduced to enzyme assay against dihydrofolate reductase (DHFR) on *E. coli* organism and DNA gyrase with two different organisms as *S. aureus* and *B. subtilis* to determine the inhibitory activities expressed as IC_50_ (µM), using Trimethoprim and Ciprofloxacin, respectively, as the reference drugs. The result is shown in Table 8 and Figure 3.

The results show that, both Schiff bases **8a** and **9b** have an inhibitory property against two different types of DNA gyrase, where IC_50_ values of Schiff base **8a** demonstrated (7.69 ± 0.23 and 15.27 ± 0.50 µM) for *S. aureus* DNA gyrase, and Schiff base **9b** revealed (10.47 ± 0.55 and 14.25 ± 0.42 µM) for *B. subtilis* DNA gyrase and both **8a** and **9b** exhibited inhibitory stronger activities than Ciprofloxacin (26.31 ± 1.64 and 29.72 ± 1.32 µM). Moreover, Schiff base **8a** (3.98 ± 0.61 µM) with pyrazole core and piperidinyl pharmacophore showed DHFR enzyme with 1.3-fold higher potential than the positive control (Trimethoprim, 5.17 ± 0.12 µM) while the Schiff base **9b** with one pyrazole moiety showed IC_50_ value (6.48 ± 0.33 µM) with (0.80 fold), respectively. 

Finally, it is noticed that designing new Schiff bases with pyrazole moiety (mono or *Bis*) with different amide and aniline derivatives in position four and three respectively, based on pyrazole nucleus prepared from cyan-acetanilide derivatives, and the reaction of amino pyrazole obtained with different aldehyde may improve the antimicrobial activity (MIC, MBC, MDRB); therefore, the enzyme inhibitory activity (DNA gyrase) and dihydrofolate reductase (DHFR) and anticancer activity against the two different cell lines, HepG-2 and MCF-7, determine the safety and selectivity on Vero cell line.

### 2.3. In Silico Studies

#### 2.3.1. Docking and Molecular Modeling Study

**Figure 4:**
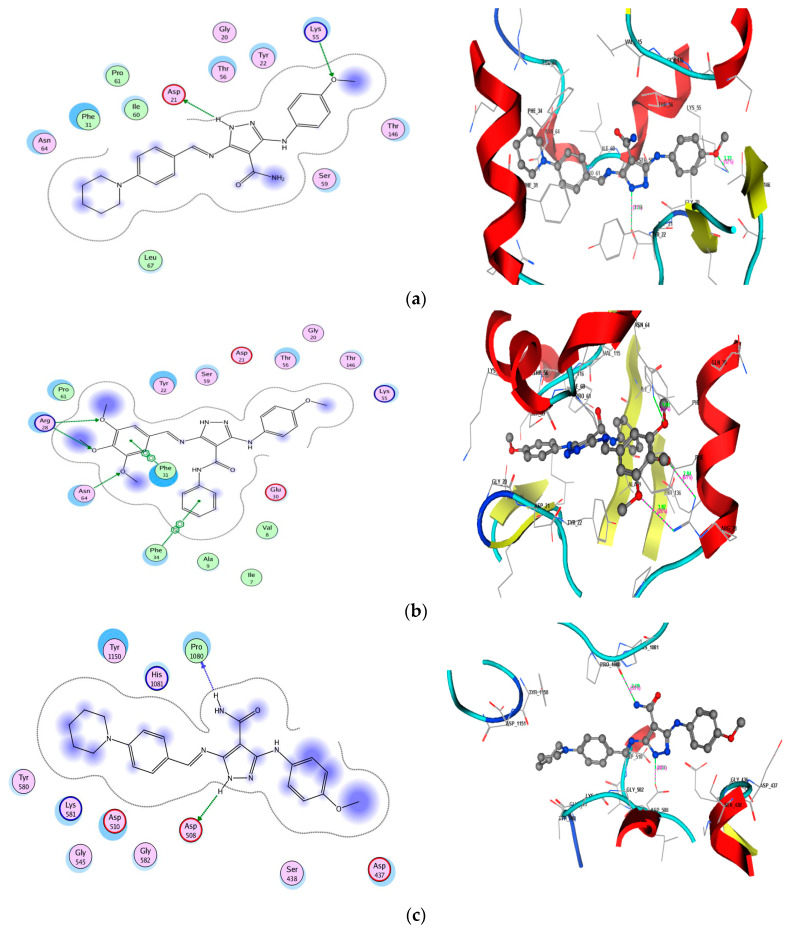

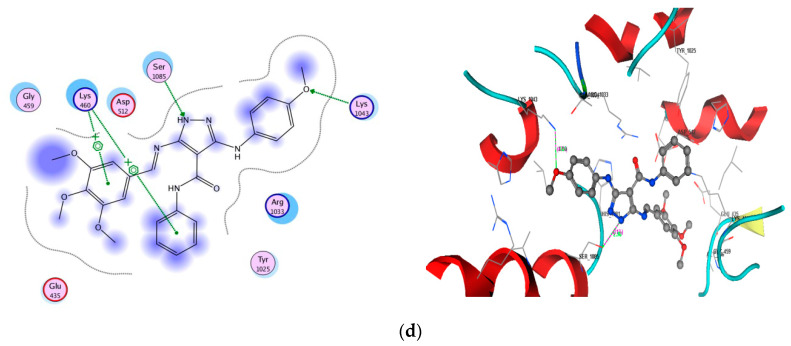
(**a**) 2D and 3D interactions of compound **8a** in the active site of 1DLS. (**b**) 2D and 3D interactions of compound **9b** in the active site of 1DLS. (**c**) 2D and 3D interactions of compound **8a** in the active site of 2XCT. (**d**) 2D and 3D interactions of compound **9b** in the active site of 2XCT.

In recent years, molecular modeling study is considered as an important study because it provides a more accurate picture of biologically active molecules at the atomic level and plays a major role in the drug designing process [67]. Docking process was performed using Molecular Operating Environment software 10.2008 (MOE), Chemical Computing Group Inc., Montreal, Quebec, Canada. Two proteins were obtained from the protein data bank (https://www.rcsb.org/) for this purpose. First one (PDB: 1DLS) for dihydrofolate reductase enzyme (DHFR) that co-crystallized with Methotrexate (MTX) [68], and another one is *S. aureus* DNA gyrase enzyme (PDB: 2XCT) and Ciprofloxacin was found co-crystallized inside the pocket [57,60]. The docking results involving all energy score (S) (Kcal/mol), binding of amino acids inside pocket with interacting groups of the ligand in docking compounds, and lengths of the bond are summarized in Table 9 and represented in Figure 4a–d.

Docking of the promising compounds **8a**, **9b** inside the active site of (PDB: 1DLS) for the dihydrofolate reductase enzyme (DHFR) revealed that compounds **8a**, **9b** provide a docking pose with an energy score (S= −18.96 and −26.13 Kcal/mol) compared to Methotrexate (MTX) (S = −27.31 Kcal/mol) that forms five hydrogen bonds, two hydrogen bond donor with Ile 7 and Glu 30 two amino of pyrimidine, and three hydrogen bond acceptor two with Arg 70 and the third one with Asn 64 with bond length (3.12, 2.68 and 2.64 Å). It was observed that the two Schiff bases **8a** and **9b** (Figure 4a,b) mainly interacts with the target enzyme by showing hydrogen bonding interaction for example, Schiff base **8a** with IC_50_ values (3.98 ± 0.61 µM) showed two hydrogen bonds with one hydrogen bond donor between Asp 21 and NH of pyrazole nucleus and another hydrogen bond acceptor between Lys 55 and the oxygen atom of the methoxy group of the *p*-anisidine amine with bond length 3.10 and 3.33 Å respectively. On the other hand, compound **9b** showed energy score near to Methotrexate (MTX), with three hydrogen bond acceptor between Arg 28 and two oxygen of methoxy groups of *tri*-methoxy benzaldehyde derivatives with (2.84 and 3.07 Å), and the third methoxy group forms a bond with Ars 64 with bond length (3.00 Å) as well as two arene-arene interaction with two amino acids Phe 34 and Phe 31, beside hydrophobic interaction.

While for *S. aureus* DNA gyrase enzyme (PDB: 2XCT), the natural ligand 1-cyclopropyl-6-fluoro-4-oxo-7-(piperazin-1-yl)-1,4-dihydroquinoline-3-carboxylic acid (Ciprofloxacin) advertised two hydrogen bond interactions with Ser 1084 amino acid with bond length 2.49, 2.60 Å *via* carboxylate ion and self-docking process showed that Ciprofloxacin binds with the pocket with an energy score (S) = −11.87 kcal/mol. Both Schiff bases **8a** and **9b** with *S. aureus* DNA gyrase IC_50_ values (7.69 ± 0.23 and 10.47 ± 0.55 µM) showed score energy (S = −19.09, −21.74 Kcal/mol) higher than Ciprofloxacin (S = −11.87 Kcal/mol), respectively (see Table 9) [57,60]. As for amino acid interactions, Schiff base 8a showed two hydrogen bond donors with Asp 508 through the NH of pyrazole, in addition to hydrogen bond between Pro 1080 and amino of amide group (Figure 4c). Schiff base **9b** formed two hydrogen bonds (Figure 4d) Lys 1043 and Ser 1085 through the oxygen of methoxy group and NH of pyrazole as well as two arene-cation interactions between phenyl derivatives of tri-substituted aldehyde and amide derivatives with Lys 460. 

Finally, all the docked compounds showed desirable interaction, especially hydrogen bond and arene-cation or arene-arene interaction with two protein targets, and that proceeds mainly through NH of pyrazole or oxygen of methoxy groups, present in our new promising compounds. It can be concluded that the newly designed pyrazole Schiff bases, mainly mono pyrazole Schiff bases **8a** and **9b** could exert their antimicrobial, anticancer activity with selectivity to the cancer cell and induced increased immunity against pathogen and tumor via two different mechanisms as DHFR and DNA gyrase inhibitors. 

#### 2.3.2. Predication of the Physicochemical and Pharmacokinetics Properties

The physicochemical properties to evaluate the drug-likeness of the two pyrazole Schiff bases (**8a** and **9b**) were generated *in silico* using Swiss ADME (http://swissadme.ch/index.php#undefined). Also, the reference drugs (Norfloxacin, Ciprofloxacin, and Trimethoprim) were generated, and the results are shown in Table 10. 

The Lipinski rule-of-five (Ro5) include four parameters: (a) MW ≤ 500 as molecular weight, (b) MLogP ≤ 4.15 as lipophilicity, (c) N or O ≤ 10 as hydrogen bond acceptors, (d) NH or OH ≤ 5 as hydrogen bond donors [69]. 

From Table 10, the number of hydrogen bond acceptors (nHBA), donors (nHBD), and lipophilicity property for the two pyrazole Schiff bases were in agreement with the Lipinski’s rule of five (Ro5). The molecular weight of the Schiff base (**9b**) is more than 500 g/mol. The pyrazole Schiff bases must not be violated more than one parameter to become an oral drug concerning bioavailability [70]. Therefore, the two pyrazole Schiff bases (**8a** and **9b**) may become an oral drug**.**

Veber (GSK) filter includes two parameters: (a) *n*RB ≤ 10 as rotatable bonds, (b) TPSA ≤ 140 as topological polar surface area [71]. According to this rule, the pyrazole Schiff base **8a** is in agreement with Veber filter. 

The pharmacokinetics properties of the two pyrazole Schiff bases (**8a** and **9b**), and the reference drugs (Norfloxacin, Ciprofloxacin, and Trimethoprim) were generated in silico using Swiss ADME (http://swissadme.ch/index.php#undefined), shown in Table 11 and we can deduce that:

- Schiff base **8a** and the three reference drugs show a high gastrointestinal absorption (GI).
- The two pyrazole Schiff bases (**8a** and **9b**) and the reference drugs did not show any effect on the central nervous system as none of the Schiff bases show blood-brain barrier (BBB) permeation.
- None of the Schiff bases are predicted as non-substrate for the permeability of glycoprotein (P-gp). Where P-glycoprotein is appraising active efflux by biological membranes and protects the central nervous system.
- The two Schiff bases (**8a** and **9b**) are predicted as inhibitors of CYP2C19, CYP2C9, CYP2D6, and CYP3A4 enzymes excluding **8a** as a non-inhibitor of CYP2D6 enzyme. Also, the two Schiff bases are predicted as non-inhibitors of CYP1A2 enzyme. The reference drugs are predicted as non- inhibitors of five enzymes.
- The log of skin permeability coefficient (log Kp) is predicted for the two Schiff bases **8a** and **9b** as −5.95 and −5.81 cm/s, respectively. The log Kp of the reference drugs ranged from −7.42 to −9.09 cm/s.

## 3. Experimental Section

### 3.1. Chemistry

Melting points were recorded on a Gallenkamp apparatus. The IR spectra were performed (KBr) on a 1650 FT-IR instrument. NMR spectra (DMSO-*d*_6_) were recorded on a Varian spectrometer (^1^H-NMR: 300/400 MHz and ^13^C-NMR: 76/101 MHz). Mass spectra and elemental analyses were determined at the Microanalytical Center, Cairo University, Egypt.

Compounds, {5-amino-*N*-aryl-3-(4-methoxyphenylamino)-1*H*-pyrazole-4-carboxamide **1a**–**d** [51,52,53], 5-chloro-3-methyl-1-phenyl-1*H*-pyrazole-4-carbaldehyde (**3**) [72] and 4-(piperidin-1-yl)benzaldehyde (**4**) [73]}, were prepared according to the reported procedure. 

1,5-Dimethyl-3-oxo-2-phenyl-2,3-dihydro-1*H*-pyrazole-4-carbaldehyde (4-antipyrinecarboxaldehyde) (**2**) and 3,4,5-trimethoxybenzaldehyde (**5**) were of Merck AR grade, Germany. 

*General method for synthesis of double pyrazole Schiff bases (**6a**–**d** and **7a**–**d**) or pyrazole Schiff bases (**8a**–**d** and **9a**–**d**):*

Compounds **1a**–**d** (0.01 mol) were mixed with 1,5-dimethyl-3-oxo-2-phenyl-2,3-dihydro-1*H*-pyrazole-4-carbaldehyde (**2**), 5-chloro-3-methyl-1-phenyl-1*H*-pyrazole-4-carbaldehyde (**3**), 4-(piperidin-1-yl)benzaldehyde (**4**), or 3,4,5-trimethoxybenzaldehyde (**5**) in absolute EtOH (25 mL) and a catalytic amount of glacial acetic acid (1 mL). The reaction mixture was refluxed for one hour; the solid product obtained on hot was filtered off and recrystallized from EtOH to afford the corresponding new *bis*-pyrazole Schiff bases **6a**–**d**, **7a**–**d** or mono-pyrazole Schiff bases **8a**–**d** and **9a**–**d**, respectively.

*5-((1,5-Dimethyl-3-oxo-2-phenyl-2,3-dihydro-1H-pyrazol-4-yl)methyleneamino)-3-(p-methoxyphenylamino)-1H-pyrazole-4-carboxamide* (**6a**): Yellow crystals; melting point: 274–276 °C; yield: 79%. IR (KBr) ν_max_/cm^−1^ 3359, 3165 (NH, NH_2_), 1675, 1654 (2C=O). ^1^H-NMR (400 MHz) δ ppm: 2.61 (s, 3H, CH_3_), 3.37 (s, 3H, NCH_3_), 3.70 (s, 3H, OCH_3_), 6.85 (d, 2H, *J* = 8.2 Hz, ArH), 7.19 (s, 1H, NH_2_-amide, exchangeable with D_2_O), 7.37 (d, 2H, *J* = 7.4 Hz, ArH), 7.45–7.48 (m, 3H, ArH), 7.56 (t, 2H, ArH), 7.80 (s, 1H, NH_2_-amide, exchangeable with D_2_O), 8.68 (s, 1H, -CH=N-), 8.85, 12.37 (2s, 2H, 2NH, exchangeable with D_2_O). ^13^C-NMR (101 MHz) δ ppm: 11.50 (CH_3_), 33.93 (NCH_3_), 55.21 (OCH_3_), 101.94, 105.97, 114.24, 117.08, 126.85, 128.42, 129.38, 133.70, 135.49, 144.36, 148.19, 153.38, 154.30, 156.79 (18C), 162.54 (C=O, amide), 166.76 (C=O, antipyrine). MS (*m*/*z*, %): 445 (M^+^, 13.44). Anal. Calcd. (%) for C_23_H_23_N_7_O_3_ (445.47): C, 62.01; H, 5.20; N, 22.01. Found: C, 62.23; H, 5.07; N, 22.16.

*5-((1,5-Dimethyl-3-oxo-2-phenyl-2,3-dihydro-1H-pyrazol-4-yl)methyleneamino)-3-(p-methoxyphenylamino)-N-phenyl-1H-pyrazole-4-carboxamide* (**6b**): Yellow crystals; melting point: 241–243 °C; yield: 82%. IR (KBr) ν_max_/cm^−1^ 3427, 3249 (NH), 1672, 1655 (2C=O). ^1^H-NMR (400 MHz) δ ppm: 2.64 (s, 3H, CH_3_), 3.39 (s, 3H, NCH_3_), 3.72 (s, 3H, OCH_3_), 6.88 (d, 2H, *J* = 9.0 Hz, ArH), 7.01 (t, 1H, ArH), 7.27 (t, 2H, ArH), 7.42–7.52 (m, 5H, ArH), 7.60 (t, 2H, ArH), 7.88 (d, 2H, *J* = 7.6 Hz, ArH), 8.77 (s, 1H, -CH=N-), 8.79, 10.43, 12.44 (3s, 3H, 3NH, exchangeable with D_2_O). ^13^C-NMR (101 MHz) δ ppm: 11.53 (CH_3_), 33.91 (NCH_3_), 55.29 (OCH_3_), 102.01, 105.94, 114.24, 121.47, 123.52, 125.27, 128.39, 129.35, 133.71, 133.84, 135.41, 144.64, 148.13, 153.60, 154.32, 156.81 (24C), 162.36 (C=O, amide), 166.71 (C=O, antipyrine). MS (*m*/*z*, %): 521 (M^+^, 28.63). Anal. Calcd. (%) for C_29_H_27_N_7_O_3_ (521.57): C, 66.78; H, 5.22; N, 18.80. Found: C, 66.61; H, 5.37; N, 18.73.

*5-((1,5-Dimethyl-3-oxo-2-phenyl-2,3-dihydro-1H-pyrazol-4-yl)methyleneamino)-3-(p-methoxyphenylamino)-N-p-tolyl-1H-pyrazole-4-carboxamide* (**6c**): Yellow crystals; melting point: 126–128 °C; yield: 87%. IR (KBr) ν_max_/cm^−1^ 3432, 3290 (NH), 1669, 1652 (2C=O). ^1^H-NMR (400 MHz) δ ppm: 2.24 (s, 3H, CH_3_), 2.62 (s, 3H, CH_3_), 3.38 (s, 3H, NCH_3_), 3.71 (s, 3H, OCH_3_), 6.88 (d, 2H, *J* = 8.7 Hz, ArH), 7.07 (d, 2H, *J* = 8.0 Hz, ArH), 7.42 (d, 2H, *J* = 7.6 Hz, ArH), 7.47–7.61 (m, 5H, ArH), 7.76 (d, 2H, *J* = 8.1 Hz, ArH), 8.79 (s, 2H, -CH=N- & NH exchangeable with D_2_O), 10.38, 12.23 (2s, 2H, 2NH, exchangeable with D_2_O). ^13^C-NMR (101 MHz) δ ppm: 11.56 (CH_3_), 21.29 (CH_3_), 33.87 (NCH_3_), 55.31 (OCH_3_), 102.04, 105.96, 114.19, 121.52, 123.49, 125.25, 128.37, 129.31, 133.73, 133.80, 135.39, 144.61, 148.11, 153.59, 154.34, 156.82 (24C), 162.34 (C=O, amide), 166.69 (C=O, antipyrine). MS (*m*/*z*, %): 535 (M^+^, 35.07). Anal. Calcd. (%) for C_30_H_29_N_7_O_3_ (535.60): C, 67.27; H, 5.46; N, 18.31. Found: C, 67.09; H, 5.38; N, 18.47.

*N-(4-Chlorophenyl)-5-((1,5-dimethyl-3-oxo-2-phenyl-2,3-dihydro-1H-pyrazol-4-yl)methyleneamino)-3-(p-methoxyphenylamino)-1H-pyrazole-4-carboxamide* (**6d**): Orange crystals; melting point: 200–202 °C; yield: 82%. IR (KBr) ν_max_/cm^−1^ 3424, 3281 (NH), 1670, 1654 (2C=O). ^1^H-NMR (400 MHz) δ ppm: 2.61 (s, 3H, CH_3_), 3.39 (s, 3H, N-CH_3_), 3.72 (s, 3H, OCH_3_), 6.88 (d, 2H, *J* = 9.1 Hz, ArH), 7.30 (d, 2H, *J* = 8.9 Hz, ArH), 7.42–7.52 (m, 5H, ArH), 7.60 (t, 2H, ArH), 7.96 (d, 2H, *J* = 8.9 Hz, ArH), 8.71 (s, 1H, -CH=N-), 8.81, 10.66, 12.59 (3s, 3H, 3NH, exchangeable with D_2_O). ^13^C-NMR (101 MHz) δ ppm: 11.52 (CH_3_), 33.86 (N-CH_3_), 55.59 (OCH_3_), 102.08, 105.92, 114.20, 121.58, 123.51, 125.26, 128.41, 129.28, 133.71, 133.78, 135.43, 144.60, 148.14, 153.61, 154.36, 156.88 (24C), 162.36 (C=O, amide), 166.72 (C=O, antipyrine). MS (*m*/*z*, %): 556 (M^+^, 14.46). Anal. Calcd. (%) for C_29_H_26_ClN_7_O_3_ (556.01): C, 62.64; H, 4.71; N, 17.63. Found: C, 62.76; H, 4.61; N, 17.69.

*5-((5-Chloro-3-methyl-1-phenyl-1H-pyrazol-4-yl)methyleneamino)-3-(p-methoxyphenylamino)-1H-pyrazole-4-carboxamide* (**7a**): Yellow crystals; melting point: 270–272 °C; yield: 74%. IR (KBr) *ν*_max_/cm^−1^ 3342, 3166 (NH, NH_2_), 1655 (C=O). ^1^H-NMR (300 MHz) *δ* ppm: 2.52 (s, 3H, CH_3_), 3.72 (s, 3H, OCH_3_), 6.87 (d, 2H, *J* = 8.0 Hz, ArH), 7.35–7.66 (m, 9H, 7H of ArH & 2H of NH_2_-amide exchangeable with D_2_O), 8.83 (s, 1H, -CH=N-), 8.88, 12.69 (2s, 2H, 2NH, exchangeable with D_2_O). Anal. Calcd. (%) for C_22_H_20_ClN_7_O_2_ (449.89): C, 58.73; H, 4.48; N, 21.79. Found: C, 58.90; H, 4.51; N, 21.64.

*5-((5-Chloro-3-methyl-1-phenyl-1H-pyrazol-4-yl)methyleneamino)-3-(p-methoxyphenylamino)-N-phenyl-1H-pyrazole-4-carboxamide* (**7b**): Yellow crystals; melting point: 228–230 °C; yield: 79%. IR (KBr) *ν*_max_/cm^−1^ 3345 (NH), 1657 (C=O). ^1^H-NMR (300 MHz) *δ* ppm: 2.59 (s, 3H, CH_3_), 3.73 (s, 3H, OCH_3_), 6.90 (d, 2H, *J* = 7.5 Hz, ArH), 7.09 (t, 1H, ArH), 7.35 (t, 2H, ArH), 7.53–7.67 (m, 9H, ArH), 8.76 (s, 1H, -CH=N-), 8.91, 9.53, 12.87 (3s, 3H, 3NH, exchangeable with D_2_O). MS (*m*/*z*, %): 525 (M^+^, 23.62). Anal. Calcd. (%) for C_28_H_24_ClN_7_O_2_ (525.99): C, 63.94; H, 4.60; N, 18.64. Found: C, 64.07; H, 4.51; N, 18.69.

*5-((5-Chloro-3-methyl-1-phenyl-1H-pyrazol-4-yl)methyleneamino)-3-(p-methoxyphenylamino)-N-p-tolyl-1H-pyrazole-4-carboxamide* (**7c**): Yellow crystals; melting point: 264–266 °C; yield: 77%. IR (KBr) *ν*_max_/cm^−1^ 3339 (NH), 1652 (C=O). ^1^H-NMR (300 MHz) *δ* ppm: 2.27 (s, 3H, CH_3_), 2.58 (s, 3H, CH_3_), 3.72 (s, 3H, OCH_3_), 6.89 (t, 1H, ArH), 7.16 (d, 2H, *J* = 8.3 Hz, ArH), 7.46 (d, 2H, *J* = 8.4 Hz, ArH), 7.52–7.67 (m, 8H, ArH), 8.78 (s, 1H, -CH=N-), 8.90, 9.46, 12.86 (3s, 3H, 3NH, exchangeable with D_2_O). Anal. Calcd. (%) for C_29_H_26_ClN_7_O_2_ (540.02): C, 64.50; H, 4.85; N, 18.16. Found: C, 64.45; H, 4.90; N, 18.10.

*5-((5-Chloro-3-methyl-1-phenyl-1H-pyrazol-4-yl)methyleneamino)-N-(p-chlorophenyl)-3-(p-methoxyphenylamino)-1H-pyrazole-4-carboxamide* (**7d**): Yellow crystals; melting point: 269–270 °C; yield: 70%. IR (KBr) ν_max_/cm^−1^ 3354 (NH), 1659 (C=O). ^1^H-NMR (300 MHz) δ ppm: 2.58 (s, 3H, CH_3_), 3.73 (s, 3H, OCH_3_), 6.75 (t, 1H, ArH), 6.90 (d, 2H, *J* = 8.8 Hz, ArH), 7.34 (d, 2H, *J* = 9.0 Hz, ArH), 7.41 (t, 2H, ArH), 7.63–7.67 (m, 4H, ArH), 7.75 (d, 2H, *J* = 8.9 Hz, ArH), 8.69 (s, 1H, -CH=N-), 9.19, 10.26, 12.73 (3s, 3H, 3NH, exchangeable with D_2_O). MS (*m*/*z*, %): 560 (M^+^, 9.06). Anal. Calcd. (%) for C_28_H_23_Cl_2_N_7_O_2_ (560.43): C, 60.01; H, 4.14; N, 17.49. Found: C, 59.95; H, 4.20; N, 17.44.

*3-(p-Methoxyphenylamino)-5-(4-(piperidin-1-yl)benzylideneamino)-1H-pyrazole-4-carboxamide* (**8a**): Orange crystals; melting point: 225–227 °C; yield: 74%. IR (KBr) *ν*_max_/cm^−1^ 3346, 3160 (NH, NH_2_), 1654 (C=O). ^1^H-NMR (300 MHz) *δ* ppm: 1.60 (s, 6H, piperidine moiety), 3.41 (s, 4H, piperidine moiety), 3.71 (s, 3H, OCH_3_), 6.85 (d, 2H, *J* = 8.6 Hz, ArH), 7.04 (d, 2H, *J* = 8.9 Hz, ArH), 7.25 (s, 1H, NH_2_-amide exchangeable with D_2_O), 7.32–7.44 (m, 3H, 2H of ArH & 1H of NH_2_-amide exchangeable with D_2_O), 7.75 (d, 2H, *J* = 8.9 Hz, ArH), 8.66 (s, 1H, -CH=N-), 8.84, 12.36 (2s, 2H, 2NH, exchangeable with D_2_O). Anal. Calcd. (%) for C_23_H_26_N_6_O_2_ (418.49): C, 66.01; H, 6.26; N, 20.08. Found: C, 66.16; H, 6.17; N, 20.00.

*3-(p-Methoxyphenylamino)-N-phenyl-5-(4-(piperidin-1-yl)benzylideneamino)-1H-pyrazole-4-carboxamide* (**8b**): Yellow crystals; melting point: 219–220 °C; yield: 87%. IR (KBr) *ν*_max_/cm^−1^ 3350 (NH), 1655 (C=O). ^1^H-NMR (300 MHz) *δ* ppm: 1.60 (s, 6H, piperidine moiety), 3.43 (s, 4H, piperidine moiety), 3.72 (s, 3H, OCH_3_), 6.89 (d, 2H, *J* = 8.1 Hz, ArH), 7.08 (t, 3H, ArH), 7.35–7.48 (m, 4H, ArH), 7.68 (d, 2H, *J* = 8.2 Hz, ArH), 7.85 (d, 2H, *J* = 8.3 Hz, ArH), 8.71 (s, 1H, -CH=N-), 8.77, 10.14, 12.56 (3s, 3H, 3NH, exchangeable with D_2_O). ^13^C-NMR (76 MHz) *δ* ppm: 23.95, 24.93, 47.65 (5C, piperidine moiety), 55.20 (-OCH_3_), 92.57, 113.73, 114.30, 117.38, 118.81, 123.10, 125.43, 129.11, 131.36, 135.13, 138.68, 151.93, 153.89, 155.91, 160.09 (22C), 163.06 (C=O). MS (*m*/*z*, %): 494 (M^+^, 32.82). Anal. Calcd. (%) for C_29_H_30_N_6_O_2_ (494.59): C, 70.42; H, 6.11; N, 16.99. Found: C, 70.50; H, 6.05; N, 17.00.

*3-(p-Methoxyphenylamino)-5-(4-(piperidin-1-yl)benzylideneamino)-N-p-tolyl-1H-pyrazole-4-carboxamide* (**8c**): Yellow crystals; melting point: 230–232 °C; yield: 82 %. IR (KBr) *ν*_max_/cm^−1^ 3352 (NH), 1660 (C=O). ^1^H-NMR (300 MHz) *δ* ppm: 1.62 (s, 6H, piperidine moiety), 2.28 (s, 3H, CH_3_), 3.46 (s, 4H, piperidine moiety), 3.72 (s, 3H, OCH_3_), 6.88 (d, 2H, *J* = 8.6 Hz, ArH), 7.11 (d, 2H, *J* = 8.9 Hz, ArH), 7.19 (d, 2H, *J* = 8.1 Hz, ArH), 7.47–7.59 (m, 4H, ArH), 7.85 (d, 2H, *J* = 8.9 Hz, ArH), 8.71 (s, 1H, -CH=N-), 8.77, 10.06 (2s, 2H, 2NH, exchangeable with D_2_O), 12.56 (s, 1H, NH). Anal. Calcd. (%) for C_30_H_32_N_6_O_2_ (508.61): C, 70.84; H, 6.34; N, 16.52. Found: C, 70.90; H, 6.25; N, 16.43.

*N-(p-Chlorophenyl)-3-(p-methoxyphenylamino)-5-(4-(piperidin-1-yl)benzylideneamino)-1H-pyrazole-4-carboxamide* (**8d**): Yellow crystals; melting point: 268–270 °C; yield: 81 %. IR (KBr) *ν*_max_/cm^−1^ 3365 (NH), 1657 (C=O). ^1^H-NMR (300 MHz) *δ* ppm: 1.62 (s, 6H, piperidine moiety), 3.45 (s, 4H, piperidine moiety), 3.72 (s, 3H, OCH_3_), 6.89 (d, 2H, *J* = 9.0 Hz, ArH), 7.11 (d, 2H, *J* = 9.2 Hz, ArH), 7.42 (d, 2H, *J* = 8.9 Hz, ArH), 7.46 (d, 2H, *J* = 9.3 Hz, ArH), 7.70 (d, 2H, *J* = 8.9 Hz, ArH), 7.85 (d, 2H, *J* = 8.9 Hz, ArH), 8.64 (s, 1H, -CH=N-), 8.80, 10.24 (2s, 2H, 2NH, exchangeable with D_2_O), 12.30 (s, 1H, NH). Anal. Calcd. (%) for C_29_H_29_ClN_6_O_2_ (529.03): C, 65.84; H, 5.53; N, 15.89. Found: C, 65.65; H, 5.48; N, 15.71.

*3-(p-Methoxyphenylamino)-5-(3,4,5-trimethoxybenzylideneamino)-1H-pyrazole-4-carboxamide* (**9a**): Yellow crystals; Melting point: 222–224 °C; Yield: 73 %. IR (KBr) *ν*_max_/cm^−1^ 3373, 3142 (NH, NH_2_), 1654 (C=O). ^1^H-NMR (300 MHz) *δ* ppm: 3.72, 3.77, 3.87 (3s, 12H, 4OCH_3_), 6.82 (d, 2H, *J* = 9.0 Hz, ArH), 6.89 (d, 2H, *J* = 8.9 Hz, ArH), 7.25–7.46 (m, 4H, 2H of ArH & 2H of NH_2_-amide exchangeable with D_2_O), 8.85 (s, 2H, -CH=N- & NH exchangeable with D_2_O), 12.12 (s, 1H, NH, exchangeable with D_2_O). ^13^C-NMR (76 MHz) *δ* ppm: 55.04, 56.11, 60.28 (4C, 4OCH_3_), 92.17, 104.26, 115.34, 121.14, 133.85, 141.73, 153.43, 154.64, 160.12 (16C), 162.51 (C=O). MS (*m*/*z*, %): 425 (M^+^, 46.11). Anal. Calcd. (%) for C_21_H_23_N_5_O_5_ (425.44): C, 59.29; H, 5.45; N, 16.46. Found: C, 59.25; H, 5.59; N, 16.54.

*3-(p-Methoxyphenylamino)-N-phenyl-5-(3,4,5-trimethoxybenzylideneamino)-1H-pyrazole-4-carboxamide* (**9b**): Yellow crystals; melting point: 221–223 °C; yield: 72 %. IR (KBr) *ν*_max_/cm^−1^ 3446, 3294 (NH), 1652 (C=O). ^1^H-NMR (300 MHz) *δ* ppm: 3.74, 3.79, 3.92 (3s, 12H, 4OCH_3_), 6.91 (d, 2H, *J* = 9.0 Hz, ArH), 7.07 (t, 1H, ArH), 7.32–7.42 (m, 6H, ArH), 7.68 (d, 2H, *J* = 7.5 Hz, ArH), 8.69 (s, 1H, -CH=N-), 9.01, 10.05, 12.64 (3s, 3H, 3NH, exchangeable with D_2_O). ^13^C-NMR (76 MHz) *δ* ppm: 55.09, 56.81, 60.62 (4C, 4OCH_3_), 92.37, 104.42, 114.19, 121.09, 126.37, 128.04, 129.28, 132.58, 137.04, 141.55, 153.73, 154.68, 161.80 (22C), 162.90 (C=O). MS (*m*/*z*, %): 500 (M^+^-1, 15.28). Anal. Calcd. (%) for C_27_H_27_N_5_O_5_ (501.53): C, 64.66; H, 5.43; N, 13.96. Found: C, 64.42; H, 5.58; N, 14.12.

*3-(p-Methoxyphenylamino)-N-p-tolyl-5-(3,4,5-trimethoxybenzylideneamino)-1H-pyrazole-4-carboxamide* (**9c**): Yellow crystals, melting point: 234–236 °C; yield: 79%. IR (KBr) *ν*_max_/cm^−1^ 3437, 3285 (NH), 1655 (C=O). ^1^H-NMR (300 MHz) *δ* ppm: 2.27 (s, 3H, CH_3_), 3.73, 3.79, 3.92 (3s, 12H, 4OCH_3_), 6.91 (d, 2H, *J* = 8.1 Hz, ArH), 7.15 (d, 2H, *J* = 8.3 Hz, ArH), 7.40–7.59 (m, 6H, ArH), 8.70 (s, 1H, -CH=N-), 8.96, 9.95, 12.64 (3s, 3H, 3NH, exchangeable with D_2_O). ^13^C-NMR (76 MHz) *δ* ppm: 21.31 (CH_3_), 55.04, 56.79, 60.65 (4C, 4OCH_3_), 91.93, 104.26, 114.17, 121.14, 126.46, 129.37, 132.54, 136.85, 141.57, 153.74, 154.40, 161.74 (22C), 162.88 (C=O). MS (*m*/*z*, %): 515 (M^+^, 24.80). Anal. Calcd. (%) for C_28_H_29_N_5_O_5_ (515.56): C, 65.23; H, 5.67; N, 13.58. Found: C, 65.15; H, 5.79; N, 13.51.

*N-(p-Chlorophenyl)-3-(p-methoxyphenylamino)-5-(3,4,5-trimethoxybenzylideneamino)-1H-pyrazole-4-carboxamide* (**9d**): Orange crystals; melting point: 213–215 °C; yield: 87%. IR (KBr) *ν*_max_/cm^−1^ 3441, 3289 (NH), 1650 (C=O). ^1^H-NMR (400 MHz) *δ* ppm: 3.73, 3.78, 3.90 (3s, 12H, 4OCH_3_), 6.90 (d, 2H, *J* = 8.9 Hz, ArH), 7.40–7.41 (m, 6H, ArH), 7.71 (d, 2H, *J* = 8.9 Hz, ArH), 8.65 (s, 1H, -CH=N-), 9.00, 10.10, 12.75 (3s, 3H, 3NH, exchangeable with D_2_O). ^13^C-NMR (101 MHz) *δ* ppm: 55.26, 56.06, 60.31 (4C, 4OCH_3_), 92.30, 106.51, 114.45, 120.55, 126.68, 128.86, 130.21, 137.56, 141.64, 153.37, 154.07, 161.91 (22C), 162.72 (C=O). MS (*m*/*z*, %): 535 (M^+^, 19.89). Anal. Calcd. (%) for C_27_H_26_ClN_5_O_5_ (535.98): C, 60.50; H, 4.89; N, 13.07. Found: C, 60.37; H, 4.99; N, 12.91.

### 3.2. Biological Evaluation (See Supplementary Material)

The inhibition zones of pyrazole Schiff bases (**6a**–**d**, **7a**–**d, 8a**–**d,** and **9a**–**d**) and the minimal inhibitory concentrations (MIC) of the potent Schiff bases (**6b**, **7b**, **7c**, **8a**, **8d**, and **9b**) were performed according to the conventional paper disk diffusion method [54,55,74]. 

The antiproliferative activities (IC_50_, µM) of the most potent Schiff bases (**6b**, **7b**, **7c**, **8a**, **8d**, and **9b**) were performed according to the 3-[4,5-dimethyl-2-thiazolyl)-2,5-diphenyl-2*H*-tetrazolium bromide (MTT) protocol [61,62,63]. 

The immunomodulatory activity of the potent Schiff bases (**6b**, **7b**, **7c**, **8a**, **8d**, and **9b**) was evaluated according to previous work [57,66].

The in vitro enzyme assay of the most promising compounds **8a** and **9b** was carried out against DNA gyrase and dihydrofolate reductase (DHFR) enzymes using Ciprofloxacin and Trimethoprim as reference drugs according to previous work [68].

### 3.3. Molecular Docking Study

Docking simulations were performed using Molecular Operating Environment (MOE) software version 2008.10, Chemical Computing Group Inc., Montreal, Quebec, Canada. The docking process methodology of new compounds was performed according to previous work [57,58,68].

## 4. Conclusions

In conclusion, a new series of mono and *bis*-pyrazole Schiff bases **6a**–**d**-**9a**–**d** were synthesized successfully and evaluated preliminary for their in vitro antimicrobial activities. From inhibition zones values, it was found that six Schiff bases (**6b**, **7b**, **7c**, **8a**, **8d**, and **9b**) displayed more than or near to the reference drugs (Tetracycline and Amphotericin B). Therefore, Schiff bases (**6b**, **7b**, **7c**, **8a**, **8d**, and **9b**) were evaluated for their minimal inhibitory, minimal bactericidal concentrations, antiproliferative activities against two cell lines (HepG-2 and MCF-7), immunomodulatory and drug resistance properties as well as antiproliferative activity against healthy non-cancer Vero cells that exhibited IC_50_ values (≥120 µM). The results of evaluation exhibited that, the two Schiff bases containing mono-pyrazole **8a** and **9b** were more potent among the Schiff bases (**6b, 7b, 7c, 8a, 8d** and **9b**) in this study. The results of enzyme assay for the two Schiff bases **8a** and **9b** against dihydrofolate reductase (DHFR) and DNA gyrase exhibited that IC_50_ values of the both Schiff bases **8a** and **9b** were stronger than Trimethoprim and Ciprofloxacin drugs, respectively. The molecular docking revealed that the two Schiff bases **8a** and **9b** showed desirable interaction, especially hydrogen bond and arene-cation or arene-arene interaction with the two protein targets 1DLS and 2XCT. Finally, the physicochemical and pharmacokinetic properties predication displayed that the two pyrazole Schiff bases may show drug-likeness and considered as candidates for the discovery or development of new drugs.

## Figures and Tables

**Table 1:** Inhibition zone (IZ) in mm ± standard deviation of Schiff bases (**6a**–**d**, **7a**–**d**, **8a**–**d,** and **9a**–**d**) and reference drugs against pathogenic microbes.

Schiff Bases and References Drugs	*Gram-Positive Bacteria*			*Gram-Negative Bacteria*			*Fungi*	
	*Bs*	*Sa*	*Ef*	*Ec*	*Pa*	*St*	*Ca*	*Fo*
**6a**	21 ± 0.4	25 ± 0.31	21 ± 0.11	24 ± 0.2	20 ± 0.15	18 ± 0.16	22 ± 0.35	13 ± 0.3
**6b**	30 ± 0.5	24 ± 0.12	29 ± 0.55	25 ± 0.81	24 ± 0.2	20 ± 0.16	22 ± 0.56	18 ± 0.15
**6c**	20 ± 0.11	23 ± 0.29	21 ± 0.54	22 ± 0.43	NA	15 ± 0.36	17 ± 0.21	9 ± 0.35
**6d**	13 ± 0.41	NA	14 ± 0.47	19 ± 0.33	12 ± 0.63	NA	13.0 ± 0.2	NA
**7a**	20 ± 0.4	19 ± 0.31	21 ± 0.11	18 ± 0.2	20 ± 0.15	18 ± 0.16	13 ± 0.35	15 ± 0.3
**7b**	28 ± 0.16	23 ± 0.55	25 ± 0.3	21 ± 0.14	24 ± 0.78	22 ± 0.12	21 ± 0.2	17 ± 0.45
**7c**	32 ± 0.22	33 ± 0.53	29 ± 0.17	30 ± 0.29	27 ± 0.73	29 ± 0.2	27 ± 0.5	22 ± 0.11
**7d**	23 ± 0.22	24 ± 0.33	22 ± 0.35	27 ± 0.3	17 ± 0.74	17 ± 0.12	20 ± 0.5	15 ± 0.14
**8a**	27 ± 0.5	26 ± 0.14	25 ± 0.33	23 ± 0.14	25 ± 0.85	23 ± 0.11	24 ± 0.3	20 ± 0.82
**8b**	15 ± 0.45	NA	12 ± 0.74	14 ± 0.21	NA	15 ± 0.2	12 ± 0.65	NA
**8c**	22 ± 0.41	17 ± 0.78	25 ± 0.14	18 ± 0.3	NA	14 ± 0.52	19 ± 0.65	NA
**8d**	24 ± 0.5	25 ± 0.77	23 ± 0.65	26 ± 0.11	21±0.2	23 ± 0.65	25 ± 0.33	21 ± 0.16
**9a**	25 ± 0.21	21 ± 0.17	19 ± 0.14	22 ± 0.18	17 ± 0.2	20 ± 0.33	22 ± 0.19	19 ± 0.55
**9b**	25 ± 0.87	21 ± 0.3	24 ± 0.35	23 ± 0.2	21 ± 0.55	23 ± 0.4	19 ± 0.25	17 ± 0.5
**9c**	22 ± 0.18	22 ± 0.34	21 ± 0.72	23 ± 0.44	NA	23 ± 0.33	21 ± 0.5	19 ± 0.28
**9d**	18 ± 0.12	16 ± 0.54	NA	15 ± 0.96	NA	12 ± 0.61	18 ± 0.2	14 ± 0.38
**Tetracycline**	25 ± 0.22	25 ± 0.11	22 ± 0.25	23 ± 0.2	20 ± 0.5	21 ± 0.55	NA	NA
**Amphotericin B**	NA	NA	NA	NA	NA	NA	22 ± 0.2	18 ± 0.32

NA: no activity (8 mm), weak activity (8–12 mm), moderate activity (12–15 mm), strong activity (>15 mm), solvent (8 mm), and *Bacillus subtilis* (Bs), *Staphylococcus aureus* (Sa), *Enterococcus faecalis* (Ef), *Escherichia coli* (Ec), *Pseudomonas aeruginosa* (Pa), *Salmonella typhi* (St), *Candida albicans* (Ca), and *Fusarium oxysporum* (Fo).

**Table 2:** Minimal inhibitory concentrations (MIC, µg/mL) and the minimum bactericidal concentrations (MBC, µg/mL) of the potent Schiff bases against bacterial pathogens.

The Potent Schiff Bases	Gram-Positive						Gram-Negative					
	*B.s*		*S.a*		*E.f*		*E.c*		*P.a*		*S.t*	
	MIC	MBC	MIC	MBC	MIC	MBC	MIC	MBC	MIC	MBC	MIC	MBC
**6b**	7.81	14.05	15.62	31.25	9.25	18.5	62.5	87.5	31.25	53.12	15.62	28.11
**7b**	5.57	10.58	1.95	3.7	3.9	6.63	7.81	12.49	31.25	59.37	3.9	6.63
**7c**	9.25	18.5	31.25	53.12	7.81	15.62	18.51	36.5	55.5	88.8	31.25	56.25
**8a**	1.95	3.9	5.57	5.57	3.9	6.63	0.97	1.94	5.57	10.58	7.81	12.49
**8d**	3.9	6.63	7.81	15.62	15.62	31.25	7.81	15.62	27.77	55.54	18.51	36.5
**9b**	4.5	9.2	7.81	15.62	3.9	7.41	7.81	14.05	15.62	31.25	5.57	10.58
**Tetracycline**	31.25	40.62	62.5	87.5	62.5	93.75	15.62	18.74	62.5	87.5	31.25	43.75

*Bacillus subtilis* (Bs), *Staphylococcus aureus* (Sa), *Enterococcus faecalis* (Ef), *Escherichia coli* (Ec), *Pseudomonas aeruginosa* (Pa), and *Salmonella typhi* (St).

**Table 3:** Minimal inhibitory concentrations (MIC, µg/mL) and the minimum fungicidal concentrations (MFC, µg/mL) of the potent Schiff bases against fungi pathogen.

The Potent Schiff Bases	*Fungi*			
	*C.a*		*F.o*	
	MIC	MFC	MIC	MFC
**6b**	31.25	53.12	55.5	88.8
**7b**	15.62	28.11	31.25	46.87
**7c**	31.25	41.65	55.54	87.5
**8a**	7.81	15.62	15.62	27.77
**8d**	9.25	17.57	31.25	56.25
**9b**	7.81	12.49	15.62	26.55
**Amphotericin B**	15.62	34.62	31.25	65.62

*Candida albicans* (Ca) and *Fusarium oxysporum* (Fo).

**Table 4:** The IC_50_ (µM) of Schiff bases (**6b**, **7b**, **7c**, **8a**, **8d**, and **9b**) against the two cancer cell lines HepG-2 and MCF-7 as well as Vero cells (ATCC CCL-81) cell lines.

The Potent Schiff Bases	IC_50_ (µM)		
	HepG-2	MCF-7	Vero
**6b**	3.42 ± 0.17	5.09 ± 0.52	343.89 ± 0.49
**7b**	7.18 ± 0.49	4.81 ± 0.25	361.22 ± 0.23
**7c**	1.22 ± 0.23	3.24 ± 0.41	213.45 ± 0.57
**8a**	2.25 ± 0.85	1.98 ± 1.55	120.55 ± 0.34
**8d**	2.63 ± 0.44	3.69 ± 0.22	241.19 ± 1.55
**9b**	1.92 ± 0.49	2.21 ± 0.36	195.94 ± 0.57
**Doxorubicin**	3.92 ± 0.50	1.94 ± 0.80	ND

** ND meaning not determined.

**Table 5:** Intracellular killing activities of Schiff bases.

The Potent Schiff Bases (at 15.62 µg/mL)	Intracellular Killing Activity %
**6b**	**88.7 ± 0.19**
**7b**	**95.4 ± 0.98**
**7c**	**72.8 ± 0.37**
**8a**	**136.5 ± 0.3**
**8d**	**49.6 ± 0.14**
**9b**	**115.2 ± 0.5**

**Table 6:** In vitro antimicrobial activities of the most promising Schiff bases against multidrug-resistant bacteria (MDRB).

The Potent Schiff Bases	Mean Diameter of Inhibition Zone (mm) against MDRB.			
	*S. aureus* ATCC 43300	*S. aureus* ATCC 33591	*E. coli* ATCC BAA-196	*P. aeruginosa* ATCC BAA-2111
**6b**	21 ± 0.21	20 ±0. 35	25 ± 0.15	21 ± 0.66
**7b**	18 ± 0.11	15 ± 0.41	20 ± 0.44	21 ± 0.33
**7c**	17 ± 0.37	21 ± 0.19	21 ± 0.3	22 ± 0.5
**8a**	26 ± 0.15	24 ± 0.12	25 ± 0.46	27 ± 0.54
**8d**	18 ± 0.5	21 ± 0.23	16 ± 0.11	25 ± 0.99
**9b**	24 ± 0.65	25 ± 0.12	22 ± 0.2	24 ± 0.88
**Norfloxacin**	25 ± 0.50	26 ± 0.5	27 ± 0.98	24 ± 0.47

**Table 7:** Minimal inhibitory concentrations (MIC, µg/mL) and minimum bactericidal concentrations (MBC, µg/mL) of the most potent Schiff bases against MDRB.

The Potent Schiff Bases	*S. aureus* ATCC 43300		*S. aureus* ATCC 33591		*E. coli* ATCC BAA-196		*P. aeruginosa* ATCC BAA-2111	
	MIC	MBC	MIC	MBC	MIC	MBC	MIC	MBC
**6b**	6.25	11.87	7.81	15.62	3.9	7.41	7.81	15.98
**7b**	7.81	15.62	8.88	15.98	9.25	18.5	7.81	15.62
**7c**	7.81	14.83	5.55	11.1	7.81	14.83	4.44	6.66
**8a**	1.95	3.9	3.9	7.8	1.95	3.9	3.9	7.8
**8d**	15.62	31.25	3.9	7.41	15.62	31.25	6.25	11.87
**9b**	2.5	4.5	1.95	3.31	6.25	11.87	5.2	9.88
**Norfloxacin**	1.25	2.81	0.78	1.56	1.57	3.53	3.13	4.69

**Table 8:** Determination of the DNA gyrase and DHFR inhibitory activities of the most promising two Schiff bases **8a** and **9b.**

Schiff Bases	IC_50_ (Mean ± SEM, µM)		
	*S. aureus* DNA Gyrase	*B. subtilis* DNA Gyrase	DHFR *E. coli*
**8a**	7.69 ± 0.23	15.27 ± 0.50	3.98 ± 0.61
**9b**	10.47 ± 0.55	14.25 ± 0.42	6.48 ± 0.33
**Ciprofloxacin**	26.31 ± 1.64	29.72 ± 1.32	---
**Trimethoprim**	---	---	5.17 ± 0.12

**Table 9:** Docking results of the promising pyrazole Schiff bases inside 1DLS and 2XCT active site.

The Promising Compounds	Energy Score (S) (Kcal/mol)	Amino Acids	Interacting Groups	Length (Å)
**MTX (1DLS)**	−27.31	Ile 7 Glu 30 Arg 70 Asn 64 Phe 34	NH_2_ of pyrimidine ring NH_2_ of pyrimidine ring Two oxygen of carboxylate Carbonyl of amide Pyrazine ring	2.89 2.73 3.12 & 2.68 2.64 --
**8a (1DLS)**	−18.96	Asp 21 Lys 55	NH of pyrazole Oxygen of methoxy group	3.10 3.33
**9b (1DLS)**	−26.13	Arg 28 Ars 64 Phe 34 Phe 31	Two oxygen of two methoxy group in tri methoxy phenyl Methoxy of tri methoxy ald. Ph of tri methoxy aldehyde Ph of amide derivatives	2.84 3.07 3.00 -- --
**Cip. (2XCT)**	−11.87	Ser 1084 Ser 1084	CO of COOH OH of COOH	2.49 2.60
**8a (2XCT)**	−19.09	Asp 508 Pro 1080	NH of pyrazole NH_2_ of amide	2.51 2.69
**9b (2XCT)**	−21.74	Lys 1043 Ser 1085 Lys 460 Lys 460	Oxygen of methoxy group NH of pyrazole Ph of tri methyl aldehyde Ph of amide derivatives	3.00 2.98 - -
**(-) indicate arene-cation interaction; (--)indicate arene-arene interaction**				

**Table 10:** In silico the physicochemical properties of the two pyrazole Schiff bases (**8a** and **9b**) and the reference drugs for Lipinski’s rule of five and Veber filter:-.

Schiff Bases and the Reference	MW	*M*LogP	*n*HBA	nHBD	*n*RB	TPSA	Violations fromLipinski’s Rule	Violations from Veber Filter
**Rule**	**<500**	**≤4.15**	**≤10**	**≤5**	**≤10**	**≤140 Å^2^**	**Yes; 0 or 1 violation**	**Yes; 0 violation**
**8a**	418.49	2.86	4	3	7	108.63	Yes; 0 violation	Yes; 0 violation
**9b**	501.53	2.73	7	3	11	119.09	Yes; 1 violation: MW > 500	No; 1 violation: Rotors > 10
**Norfloxacin**	319.33	1.04	5	2	3	74.57	Yes; 0 violation	Yes; 0 violation
**Ciprofloxacin**	331.34	1.28	5	2	3	74.57	Yes; 0 violation	Yes; 0 violation
**Trimethoprim**	290.32	0.41	5	2	5	105.51	Yes; 0 violation	Yes; 0 violation

**Table 11:** *In silico* the pharmacokinetics properties of the two pyrazole Schiff bases (**8a** and **9b**) and the reference drugs:-.

Schiff Basesand the Reference.	GI Absorption	BBB Permeant	P-gp Substrate	CYP1A2 Inhibitor	CYP2C19 Inhibitor	CYP2C9 Inhibitor	CYP2D6 Inhibitor	CYP3A4 Inhibitor	Log Kp (cm/s)
**8a**	High	No	No	No	Yes	Yes	No	Yes	−5.95
**9b**	Low	No	No	No	Yes	Yes	Yes	Yes	−5.81
**Norfloxacin**	High	No	Yes	No	No	No	No	No	−8.98
**Ciprofloxacin**	High	No	Yes	No	No	No	No	No	−9.09
**Trimethoprim**	High	No	Yes	No	No	No	No	No	−7.42

## Supplementary Materials

Supplementary Text: Biological Evaluation.
