# Supplementary material for: Discovery of New Schiff Bases Tethered Pyrazole Moiety: Design, Synthesis, Biological Evaluation, and Molecular Docking Study as Dual Targeting DHFR/DNA Gyrase Inhibitors with Immunomodulatory Activity
Source: Molecules. 2020 Jun 2;25(11):2593. doi: 10.3390/molecules25112593 (PMC7321065; doi:10.3390/molecules25112593)
Supplement: Supplementary file 1 [file molecules-25-02593-s001.pdf]

SUPPLEMENTARY MATERIAL

For

Discovery of New Schiff Bases Tethered Pyrazole Moiety: Design, Synthesis, Biological Evaluation and Molecular Docking Study as Dual Targeting DHFR/DNA Gyrase Inhibitors with Immunomodulatory Activity

Ashraf S. Hassan[1,*], Ahmed A. Askar[2,*], Ahmed M. Naglah[3,4], Abdulrahman A. Almehizia[3,5] and Ahmed Ragab[6,*]

[1] Organometallic and Organometalloid Chemistry Department, National Research Centre, Dokki 12622 Cairo, Egypt
[2] Botany and Microbiology Department, Faculty of Science (Boys), Al-Azhar University, Nasr city, Cairo, Egypt
[3] Department of Pharmaceutical Chemistry, Drug Exploration and Development Chair (DEDC), College of Pharmacy, King Saud University, Riyadh 11451, Saudi Arabia; anaglah@ksu.edu.sa (A.M.N.); mehizia@ksu.edu.sa (A.A.A.)
[4] Peptide Chemistry Department, National Research Centre, Dokki 12622, Cairo, Egypt
[5] Department of Pharmaceutical Chemistry, College of Pharmacy, King Saud University, Riyadh 11451, Saudi Arabia
[6] Chemistry Department, Faculty of Science (Boys), Al-Azhar University, Nasr city, Cairo, Egypt
* Correspondence: ashraf_salmoon@yahoo.com (A.S.H.); drahmed_askar@azhar.edu.eg (A.A.A.); Ahmed_ragab@azhar.edu.eg (A.R.); Tel.: +20-100-664-5444 (A.S.H.); +20-101-081-5102 (A.A.A.); +20-100-934-1359 (A.R.)

Biological Evaluation

In Vitro Antimicrobial Activities

The antimicrobial activities inhibition zone (IZ, mm±standard deviation) of pyrazole Schiff bases (**6a-d**, **7a-d, 8a-d** and **9a-d**) was measurement according to the agar plate diffusion method. Briefly, 100 µl of the test bacteria/fungi were grown in 10 mL of fresh media until they reached a count of approximately 108 cells/ml for bacteria or 105 cells/mL for fungi. One mL of each sample (at 0.5 mg/mL) was added to each well (10 mm diameter holes cut in the agar gel). The plates were incubated for 24 h at 37 ^{o}C (for bacteria and yeast) and for 72 h at 27 ^{o}C (for filamentous fungi), each test was repeated three times. After incubation, the microorganism's growth was observed. Tetracycline was used as standard antibacterial drugs while Amphotericin B was used as standard antifungal drug. The resulting inhibition zone diameters were measured in millimeters and used as criterion for the antimicrobial activity. Solvent controls (DMSO) were included in every experiment as negative controls. DMSO was used for dissolving the tested compounds and showed no inhibition zones, confirming that it has no influence on growth of the tested microorganisms.

The MIC, MBC and MFC of the potent Schiff bases (6b, 7b, 7c, 8a, 8d and 9b):

The minimum inhibitory concentration (MIC), the minimum bactericidal concentration (MBC, µg/ml) and the minimum fungicidal concentration (MFC, µg/ml) of the most potent Schiff bases **(6b, 7b, 7c, 8a, 8d** and **9b)** were determined by the conventional technique termed paper disk diffusion, by applying paper disk (266812 W. Germany 12.7 mm in diameters). Bacteria were grown on nutrient agar medium, while fungi and yeast were grown on Sabouraud agar medium. The purified streptomycin was dissolved in water and loaded on paper disks with different concentrations as the following (250, 125, 62.50, 31.25, 15.63, 7.81, 3.90, 1.95, 0.98, 0.49, 0.24 and 0.12 µg/mL). Drying disks were loaded on surface of agar plates inoculated with test organism. Growth inhibition was examined after 24 hr. from incubation at 37 ^{o}C for bacteria and after 72 hr. incubation at 27 ^{o}C for fungi and yeast. Each test was repeated three times. MIC was expressed as the lowest concentration inhibiting test organism's growth.

The antiproliferative activities of the most potent Schiff bases (6b, 7b, 7c, 8a, 8d and 9b)

MTT cytotoxicity assay

3-[4, 5-Dimethyl-2-thiazolyl)-2, 5-diphenyl-2*H*-tetrazolium bromide (MTT) protocol is based on the cleavage of the tetrazolium salt by mitochondrial dehydrogenases in viable cells. Cells were dispensed in a 96 well sterile microplate (5 x 10^4 cells/well), and incubated at 37 ^{o}C with series of different concentrations, in DMSO, of each tested compound or Doxorubicin® (positive control) for 48 h in a serum free medium prior to the MTT assay. After incubation, media were carefully removed, 40 µL of MTT (2.5 mg/mL) were added to each well and then incubated for an additional 4 h. The purple formazan dye crystals were solubilized by the addition of 200 µL of DMSO. The absorbance was measured at 590 nm using a SpectraMax® Paradigm® Multi-Mode microplate reader. The relative cell viability was expressed as the mean percentage of viable cells compared to the untreated control cells.

Statistical analysis

All experiments were conducted in triplicate and repeated in three different days. All the values were represented as mean ± SD. IC_{50}s were determined by probit analysis using SPSS software program (SPSS Inc., Chicago, IL).

Immunomodulatory activity:-

Isolation of neutrophils

A 15 mL peripheral blood sample was taken from healthy volunteers collected in preservative-free heparin and 2 mL of 4.5 % dextran B in saline was added. After gently shaking, the mixture was allowed to stand for 60 min at 37 °C to sediment erythrocytes. Neutrophils were isolated by centrifugation. After removal of the residual erythrocytes by hypotonic lysis, the neutrophils were washed with Hank's balanced salt solution (HBSS) (Sigma, St Louis, USA) and then suspended at a final concentration of 25 × 10^6 cells/mL in HBSS for intracellular killing activity. The viability of neutrophils was tested by trypan blue exclusion and was greater than 90%.

Intracellular killing activities

The intracellular killing activity was measured by nitroblue tetrazolium (NBT) reduction test by the modified method**.** Isolated neutrophils were incubated in HBSS with latex particles, NBT (Sigma, St Louis, USA) then the reduced dye, blue formazon was extracted with pyridine and measured spectrophotometrically at 515 nm. The results were compared with the negative control contains all the reagents except the neutrophil suspension. The difference in the absorbance between the cultures of cells that actively phagocyte latex particles and the negative control was considered as an index of intracellular killing activity of neutrophils. Activity of the samples was calculated as the percentage ratio of the compounds in latex to positive control which is latex.

Drug resistance

The inhibition zone (IZ, mm±standard deviation) of pyrazole Schiff bases (**6b, 7b, 7c, 8a, 8d** and **9b**) was measurement according to the agar plate diffusion method against a panel of multidrug-resistant bacteria (MDRB). Also, the minimum inhibitory concentration (MIC) and the minimum bactericidal concentration (MBC, µg/ml) were determined by the conventional technique termed paper disk diffusion, by applying paper disk (266812 W. Germany 12.7 mm in diameters).

Experimental of Molecular docking study

Docking simulations were performed using Molecular Operating Environment (MOE) software version 2008.10. The most active compounds **8a**, **9b** were drawn by chem draw 2014, then exported to MOE. Energy minimization using the MMFF94x force field was calculated for each molecule. The crystal structure of both dihydrofolate reductase enzyme (DHFR) that co-crystallized with Methotrexate (MTX) and *S. aureus* DNA gyrase in complex with Ciprofloxacin were downloaded from the protein data bank (PDB ID: 1DLS, 2XCT), alpha trigonal and trigonal matcher placement methods respectively, were selected for the previous two protein and London dG scoring function were used in the docking protocol. The docking process methodology was first validated by redocking original ligand that co-crystalized inside active site and the docking of new compounds performed according to previous work.

Docking Figuer

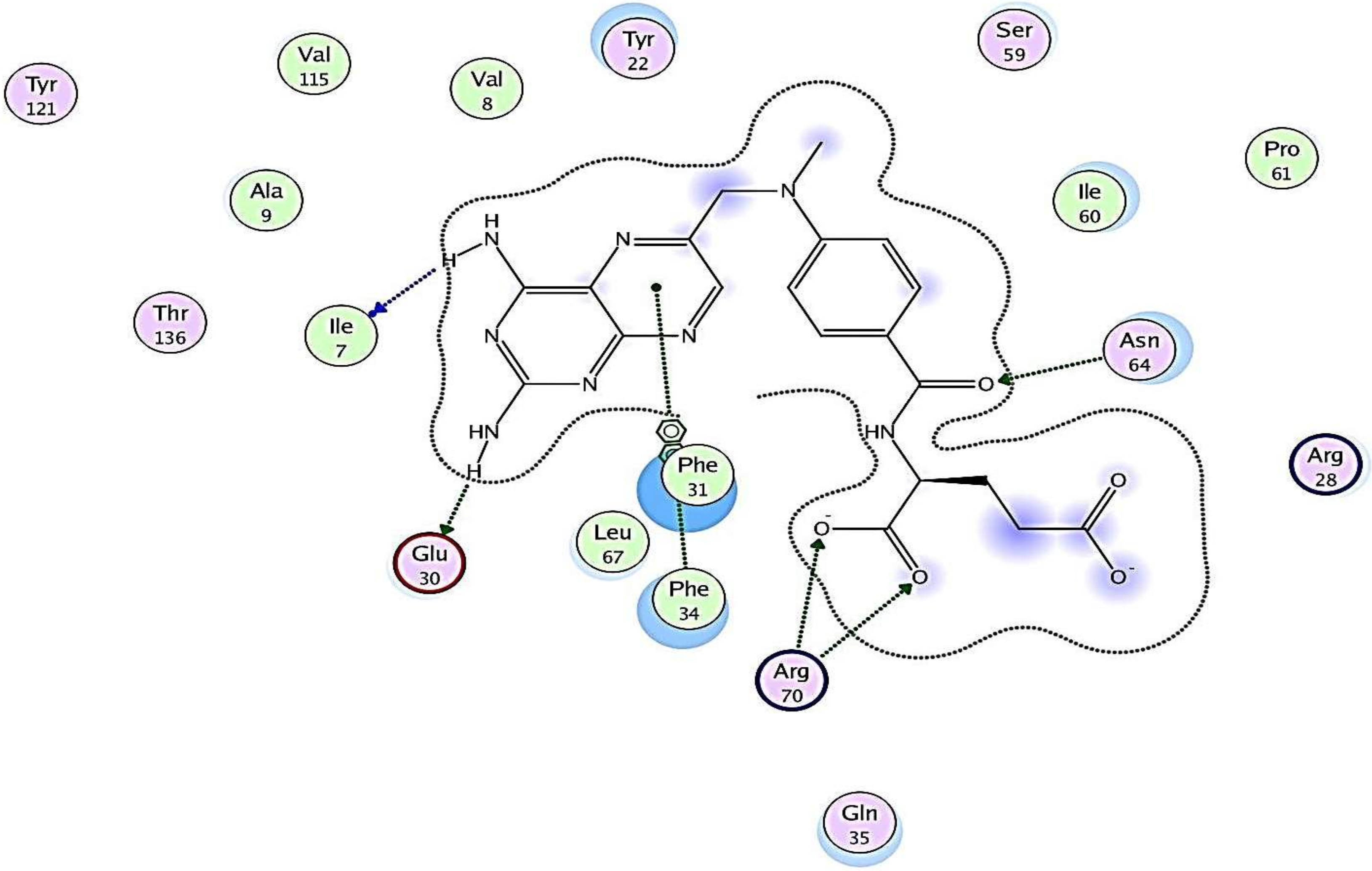

2D interactions of Methotrexate (MTX) in the active site of 1DLS

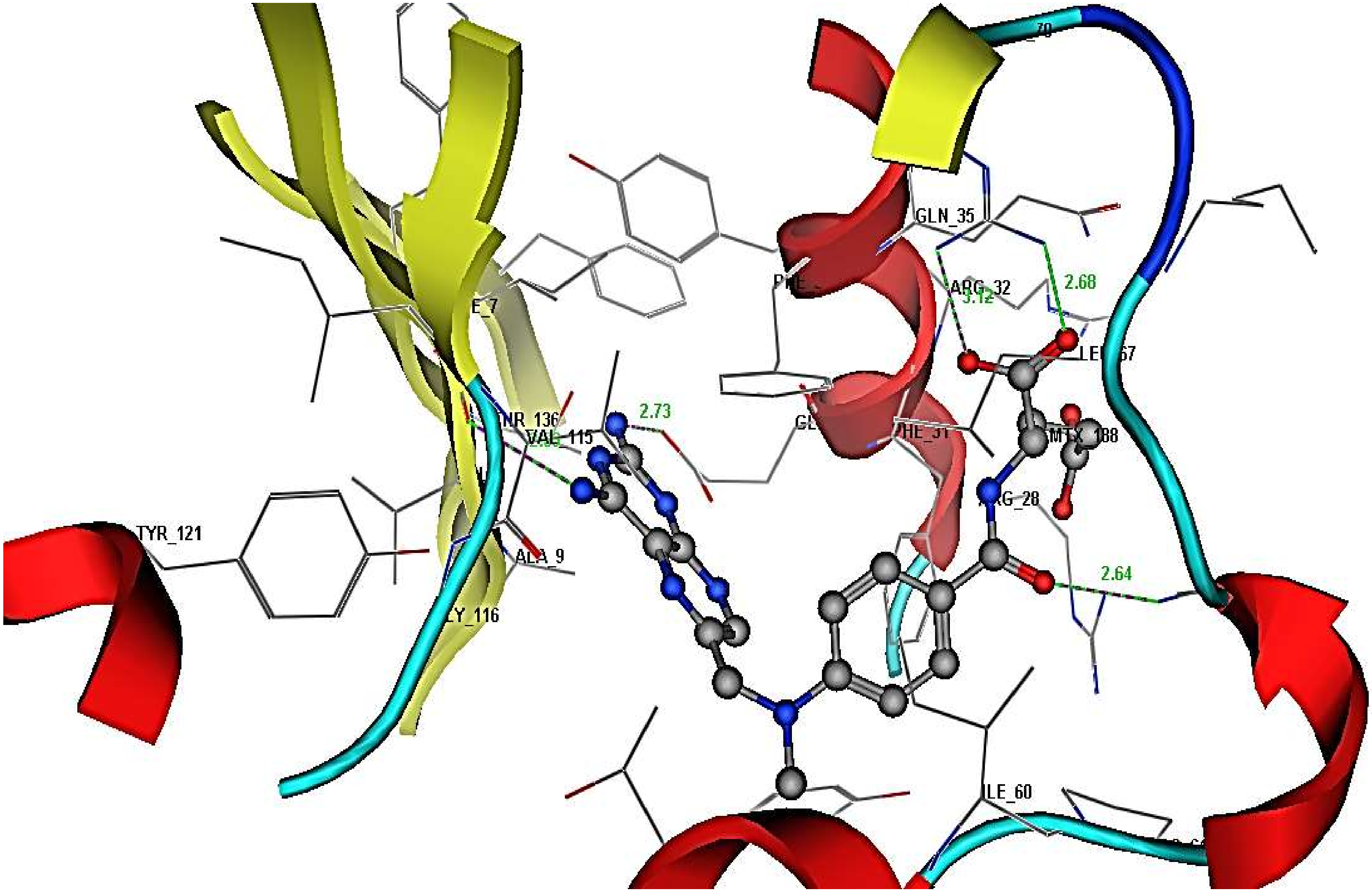

3D interactions of Methotrexate (MTX) in the active site of 1DLS

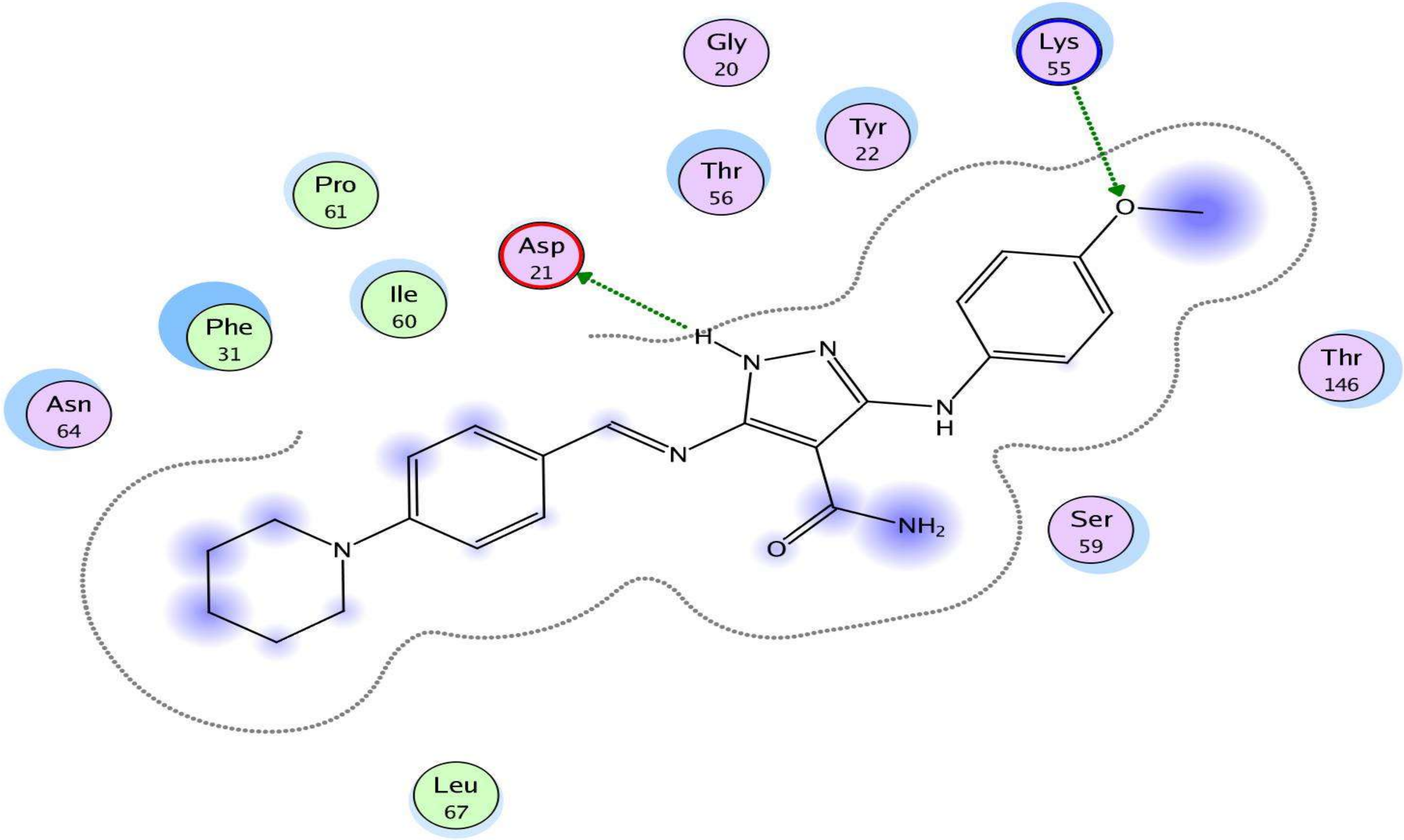

2D interactions of compound **8a** in the active site of 1DLS

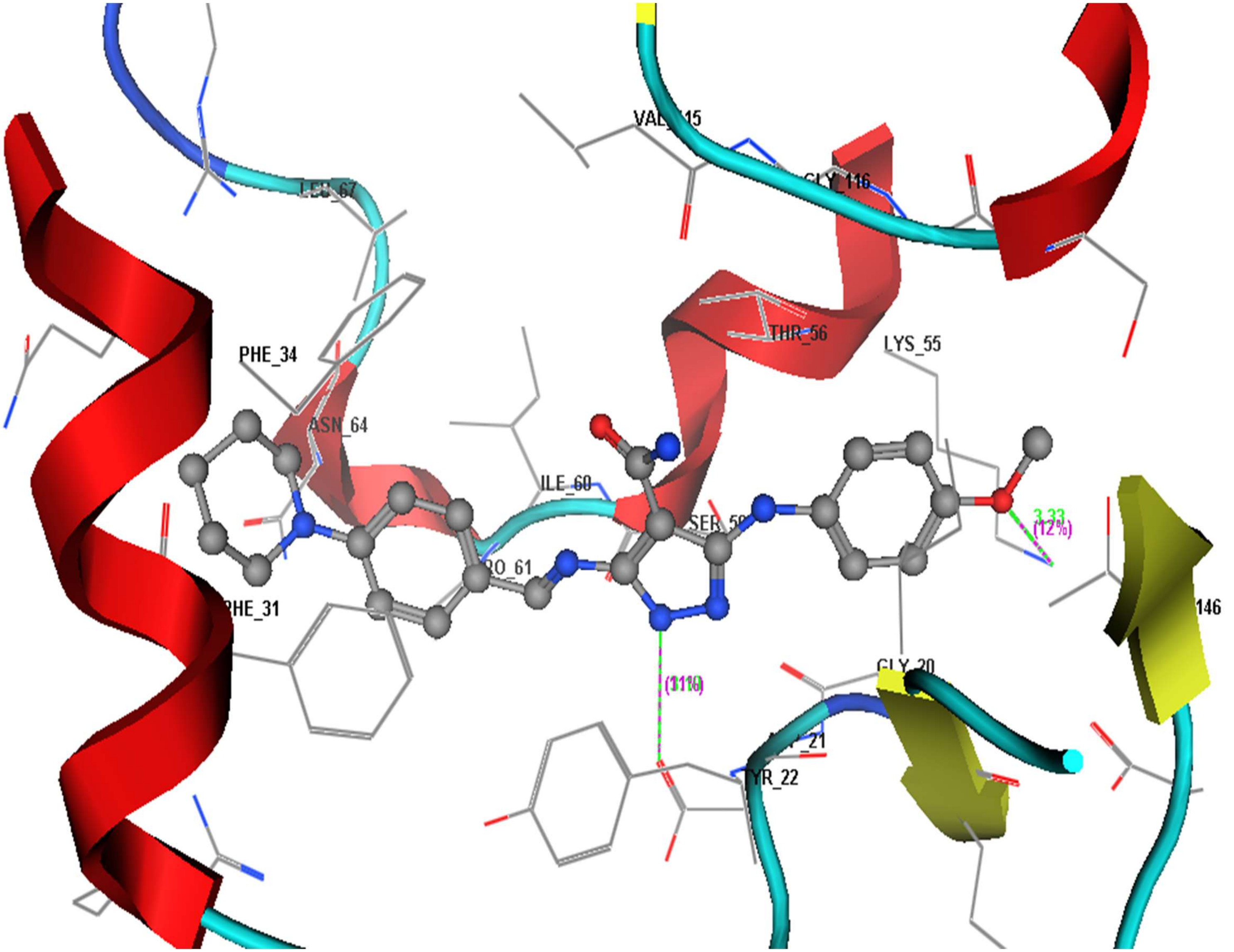

3D interactions of compound **8a** in the active site of 1DLS

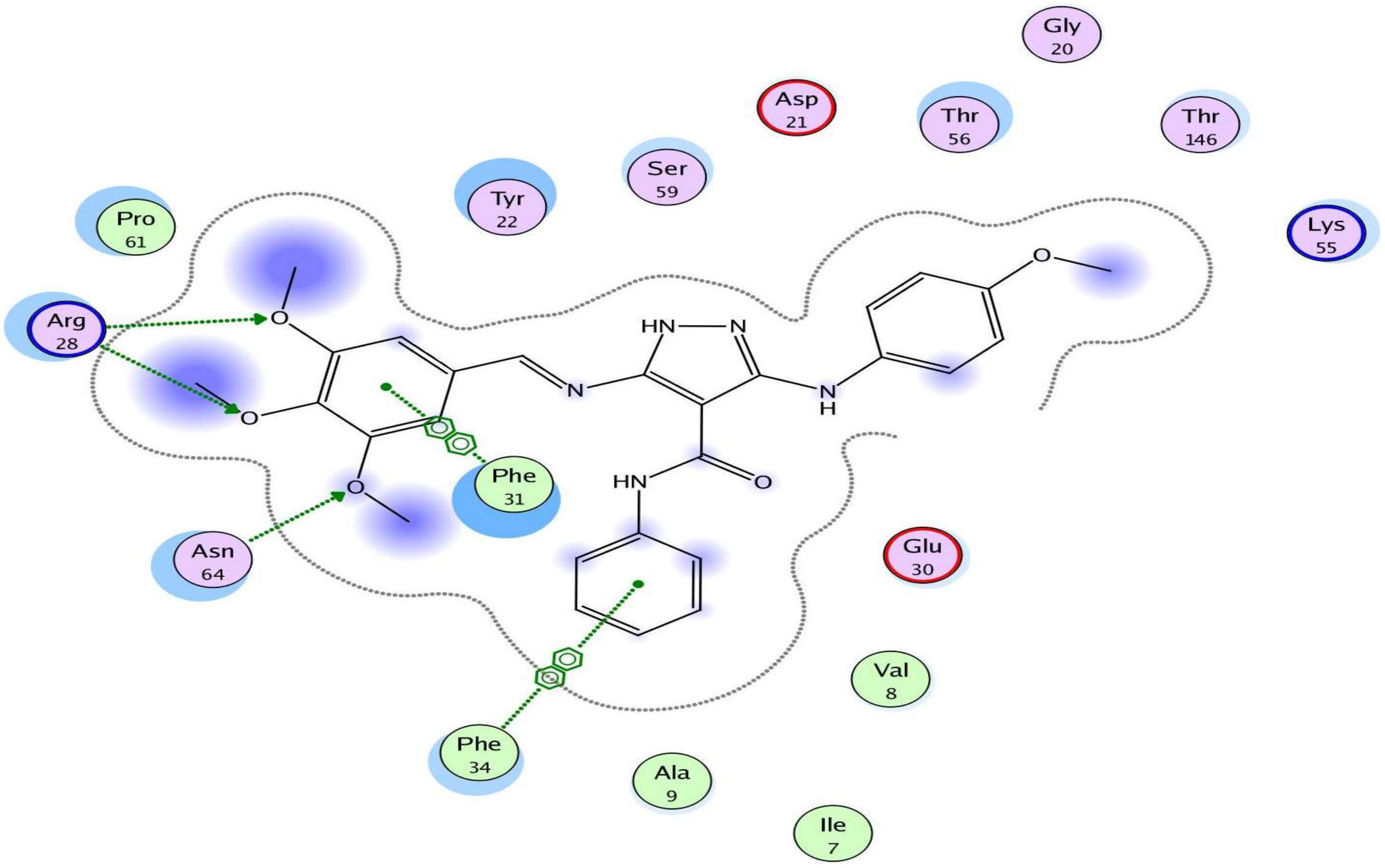

2D interactions of compound **9b** in the active site of 1DLS

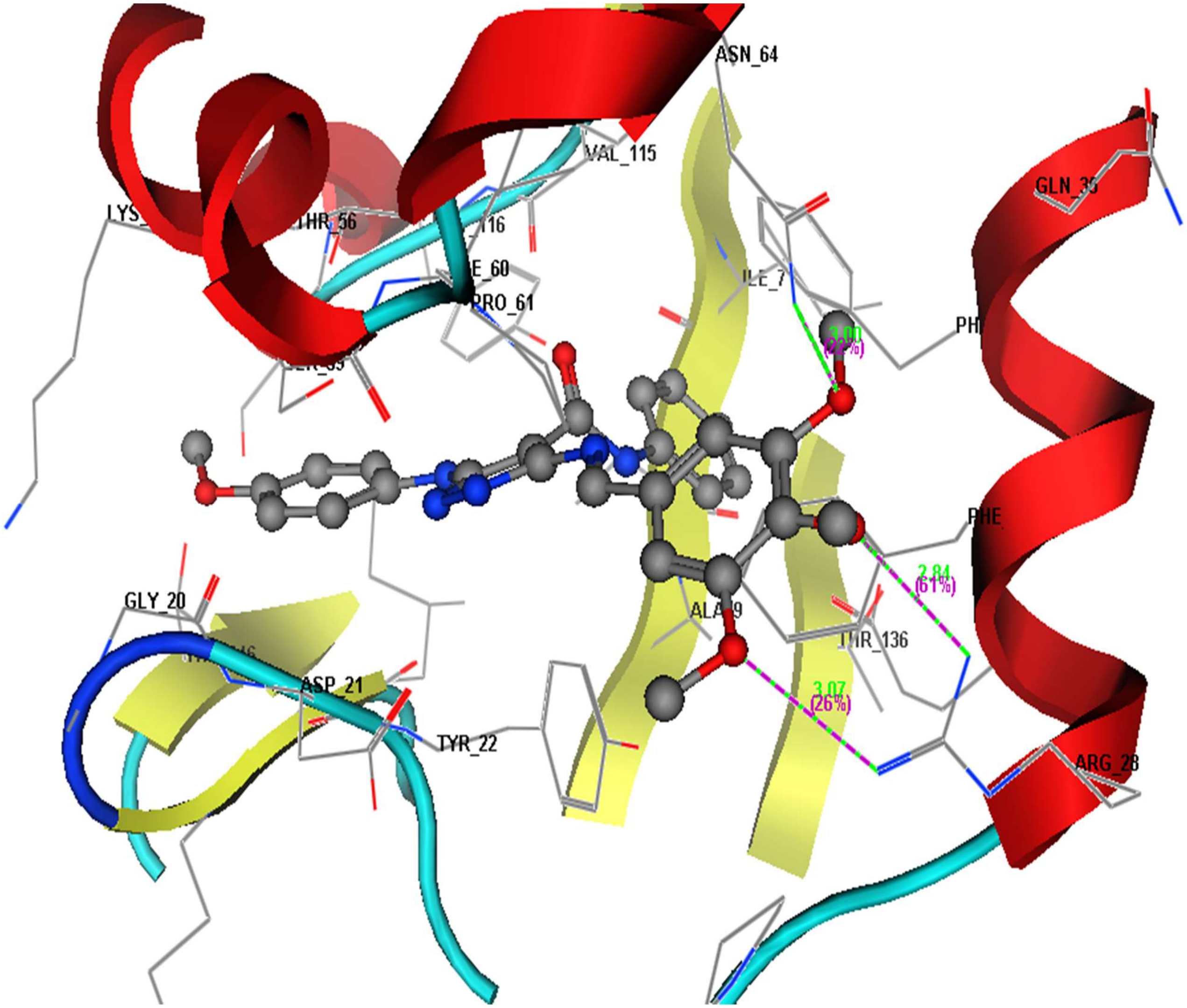

3D interactions of compound **9b** in the active site of 1DLS

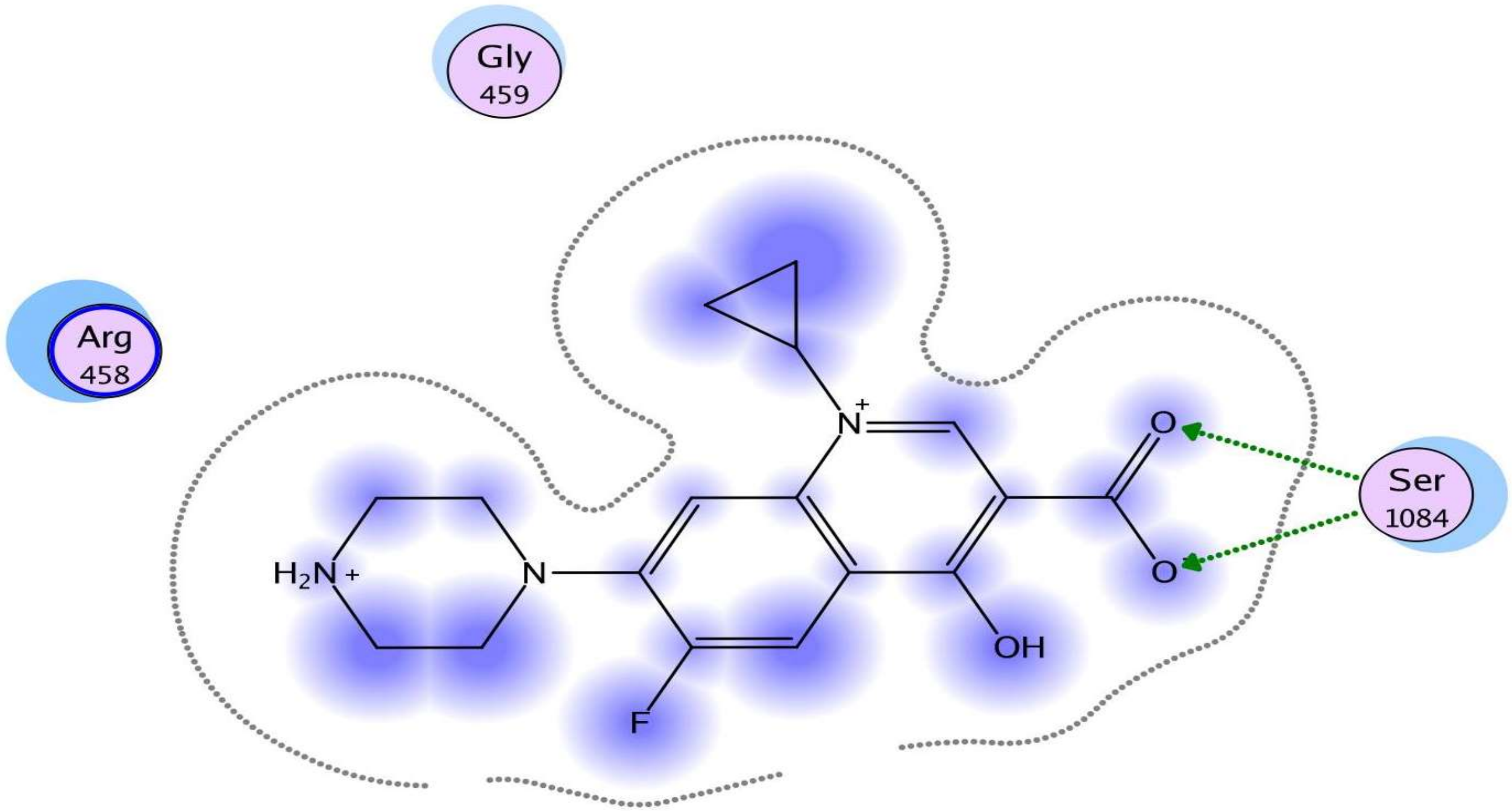

2D interactions of Ciprofloxacin in the active site of 2XCT

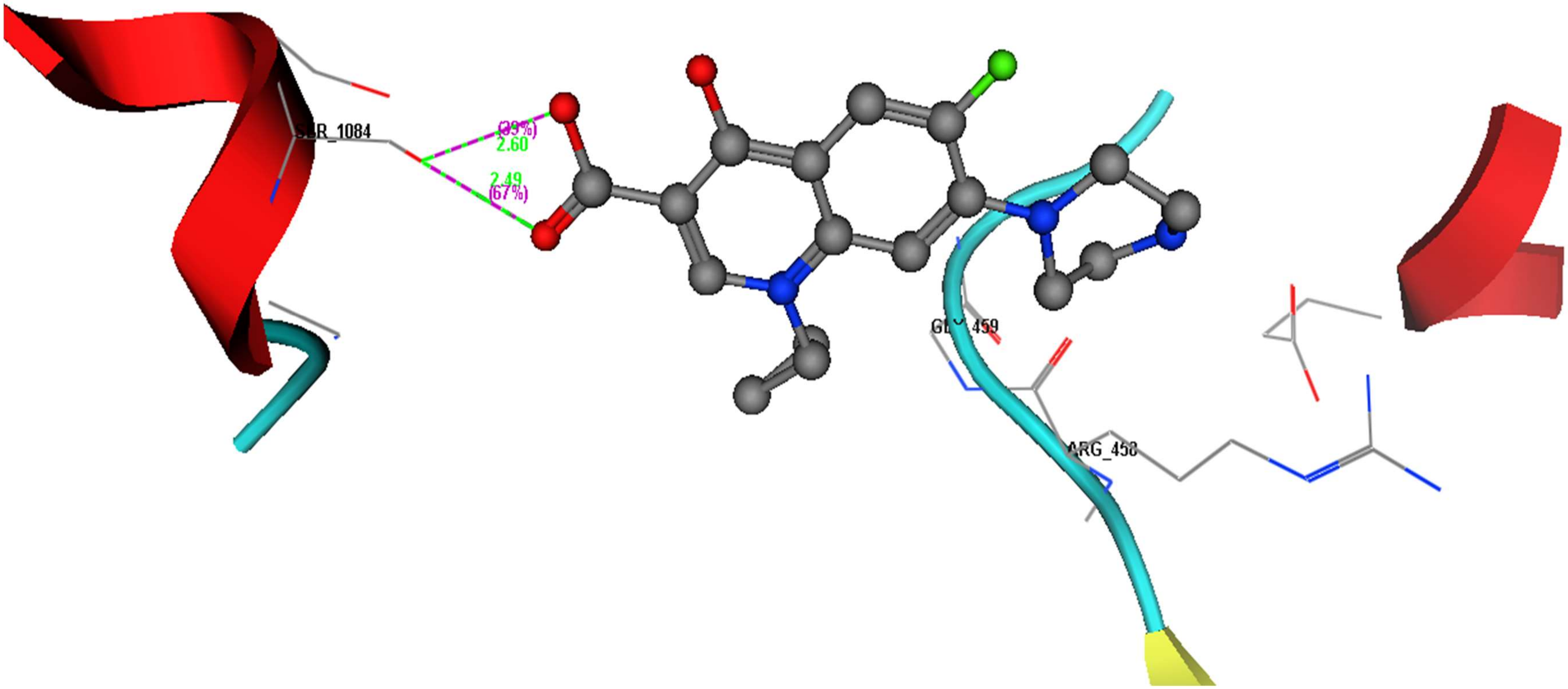

3D interactions of Ciprofloxacin in the active site of 2XCT

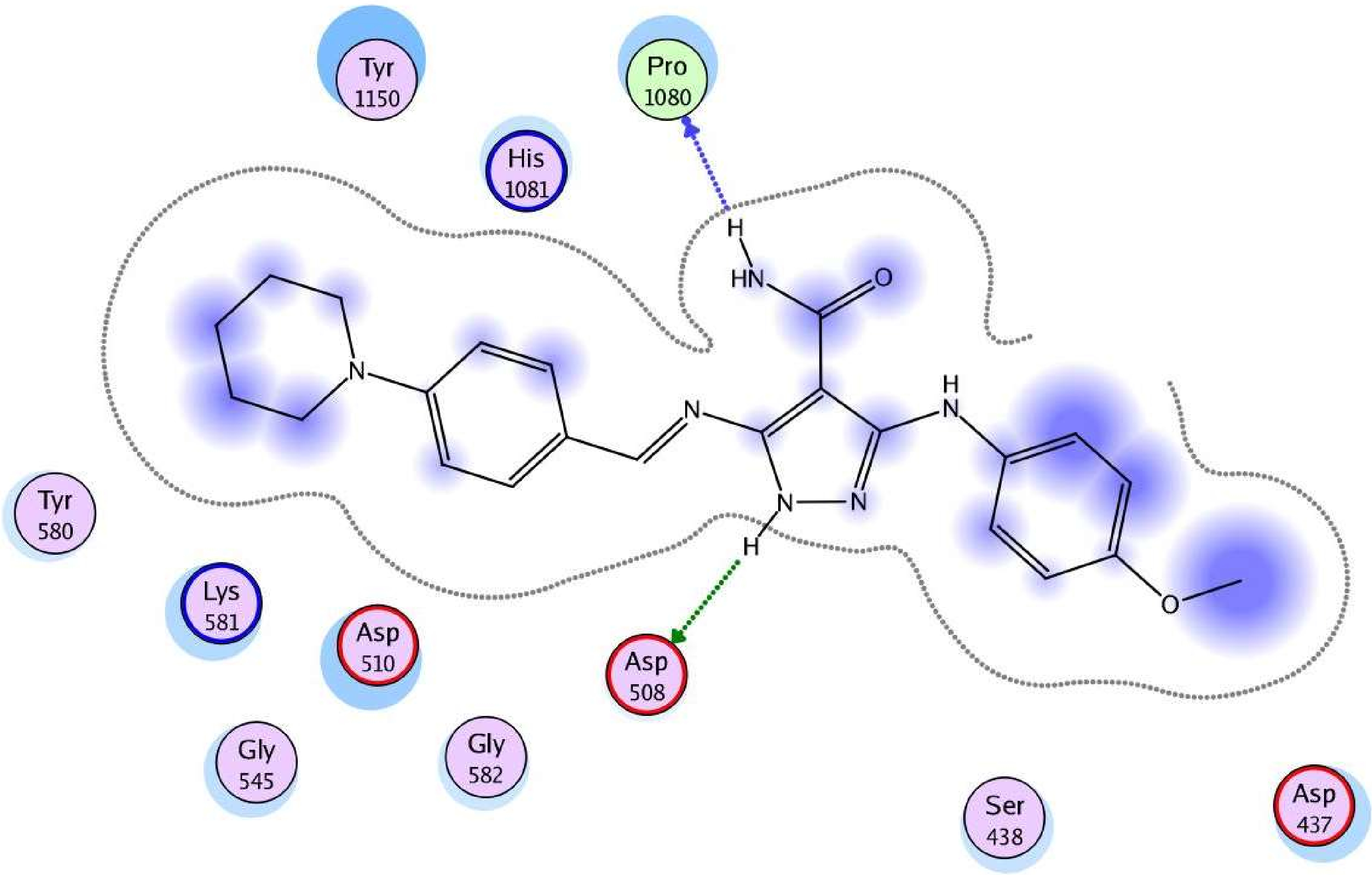

2D interactions of compound **8a** in the active site of 2XCT

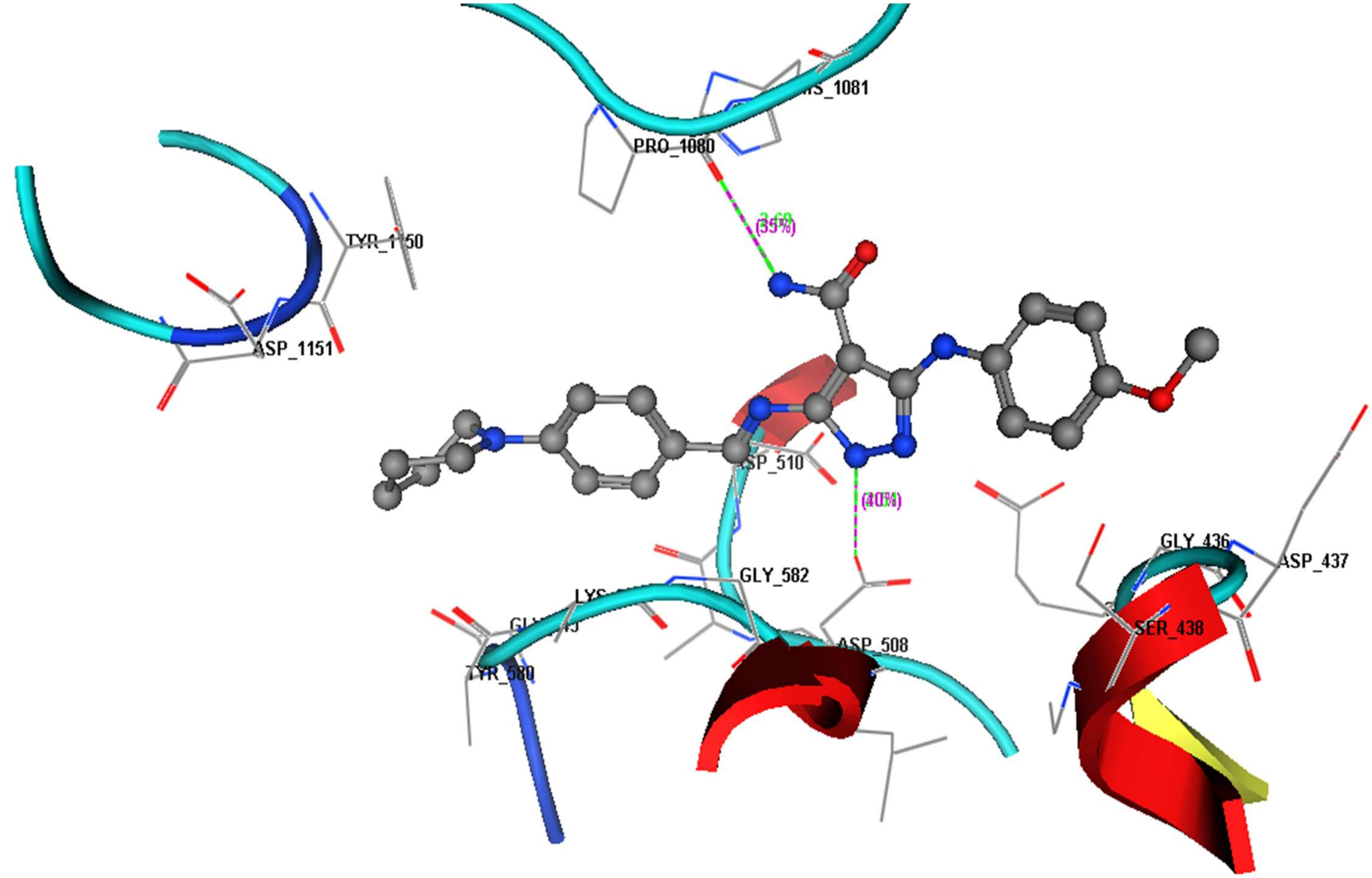

3D interactions of compound **8a** in the active site of 2XCT

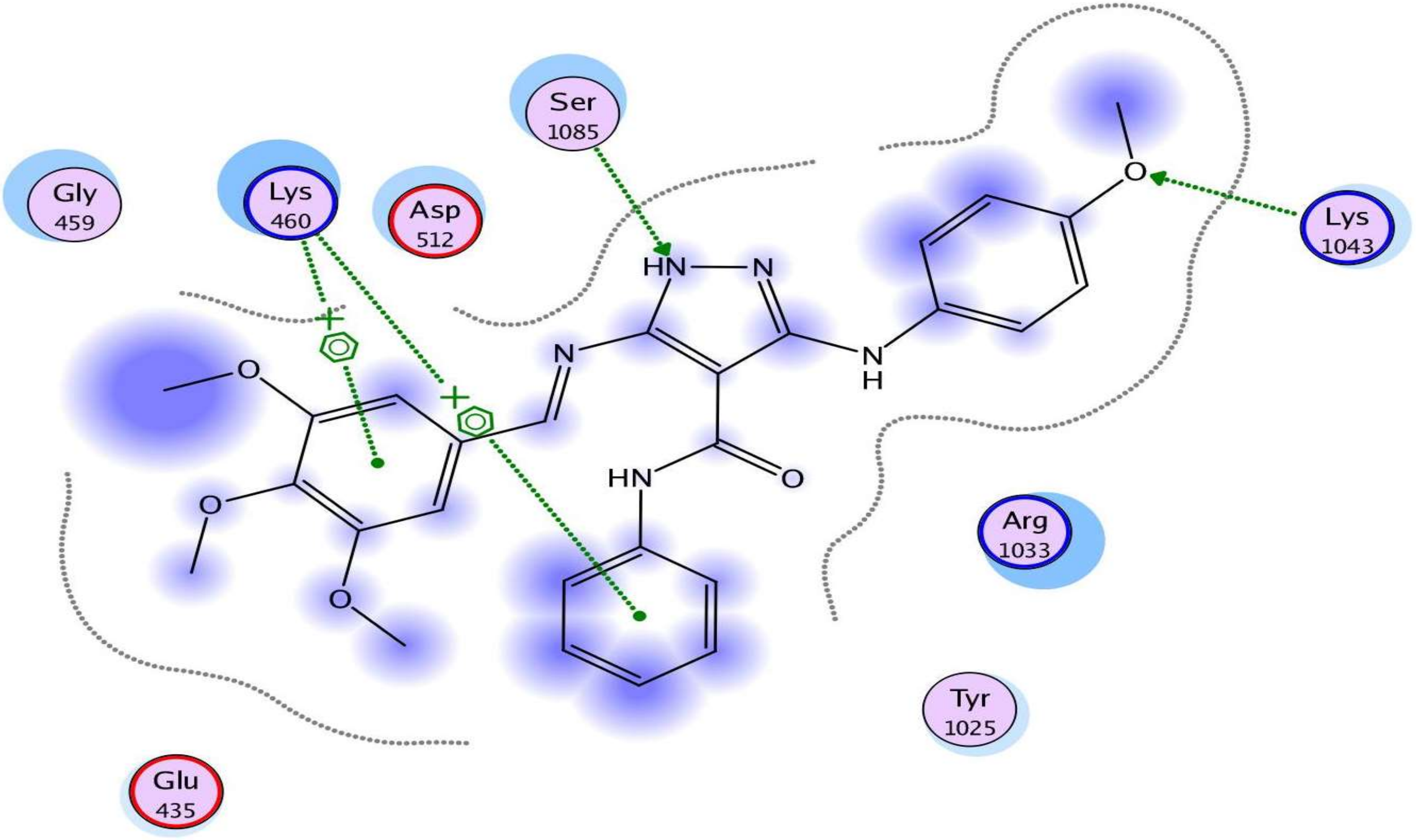

2D interactions of compound **9b** in the active site of 2XCT

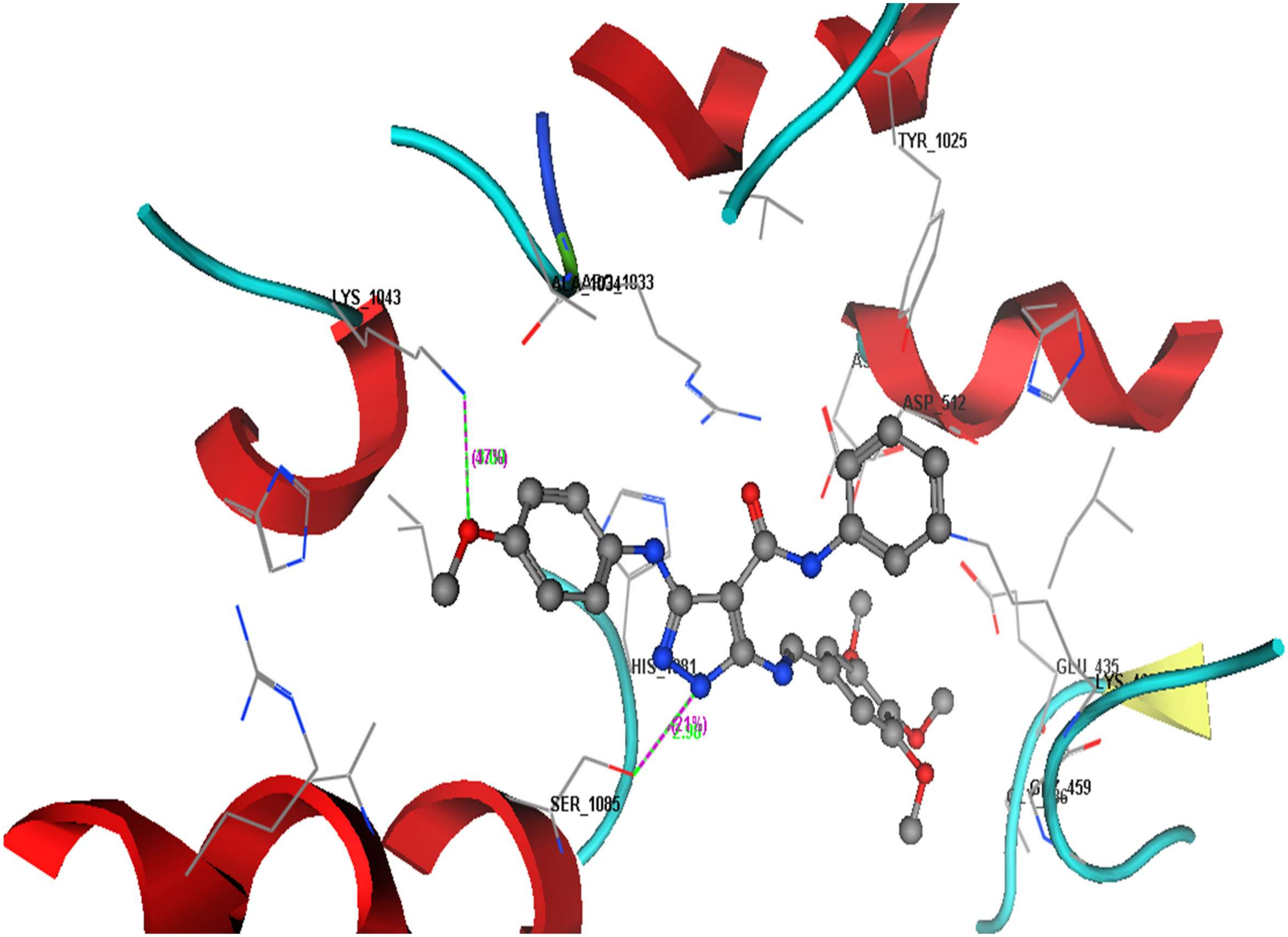

3D interactions of compound **9b** in the active site of 2XCT
